# Dorsomedial striatal neuroinflammation causes excessive goal-directed action control by disrupting astrocyte function

**DOI:** 10.1038/s41386-025-02247-4

**Published:** 2025-09-27

**Authors:** Arvie Rodriguez Abiero, Joanne M. Gladding, Jacqueline A. Iredale, Hannah R. Drury, Elizabeth E. Manning, Christopher V. Dayas, Amolika Dhungana, Kiruthika Ganesan, Karly Turner, Serena Becchi, Michael D. Kendig, Christopher Nolan, Bernard Balleine, Alessandro Castorina, Louise Cole, Kelly J. Clemens, Laura A. Bradfield

**Affiliations:** 1 School of Life Sciences, Faculty of Science, University of Technology Sydney, Sydney, NSW Australia; 2 Centre for Neuroscience and Regenerative Medicine, St. Vincent’s Centre for Applied Medical Research, St. Vincent’s Health Network, Sydney, NSW Australia; 3 School of Psychology, Faculty of Science, University of Sydney, Sydney, NSW Australia; 4 School of Biomedical Sciences and Pharmacy, College of Health, Medicine and Wellbeing, University of Newcastle, Callaghan, NSW Australia; 5 Brain Neuromodulation Research Program, Hunter Medical Research Institute, New Lambton Heights, Sydney, NSW Australia; 6 School of Psychology, University of New South Wales, Sydney, NSW Australia; 7 Decision Neuroscience Laboratory, School of Psychology, University of New South Wales, Sydney, NSW Australia; 8 Teva Pharmaceuticals, Sydney, NSW Australia

**Keywords:** Reward, Motivation

## Abstract

Compulsive actions are typically thought to reflect the dominance of habits over goal-directed action. To investigate this, we mimicked the striatal neuroinflammation that is frequently exhibited in individuals with compulsive disorders in rats, by injecting the endotoxin lipopolysaccharide into the posterior dorsomedial striatum, and assessed the consequences for behavioural control. Surprisingly, this manipulation caused rats to acquire and maintain goal-directed actions under conditions that would otherwise produce habits. Immunohistochemical analyses indicated that these behaviours were a result of astrocytic proliferation. To probe this further, we chemogenetically activated the Gi-pathway in striatal astrocytes, which altered the firing properties of nearby medium spiny neurons and modulated goal-directed action control. Together, results show that striatal neuroinflammation is sufficient to bias action selection toward excessive goal-directed control via dysregulated astrocyte function. If translatable, our findings suggest that, contrary to conventional views, individuals with striatal neuroinflammation might be more prone to maladaptive goal-directed actions than habits, and future interventions should aim to restore appropriate action control.

## Introduction

Striatal neuroinflammation is a core neuropathological feature of mental health disorders that feature compulsivity, such as obsessive compulsive disorder (OCD) and substance use disorder (SUD) [1–4]. Individuals with these disorders perform actions repetitively, often against their desires and despite negative consequences. This has led to the prevailing hypothesis that compulsions arise from a disruption to goal-directed actions and an overreliance on habits [5–8]. However, a recent article [9] challenged this view, suggesting that SUD is more aligned with goal-directed control, an assertion that has prompted much debate within the field [10–12]. To distinguish between these competing hypotheses, we here attempt reconcile this contradiction at the level of neural mechanism, by investigating how inducing striatal neuroinflammation in rats alters the balance of action control.

The neural circuits of goal-directed and habitual actions have been extensively investigated over the last three decades, with considerable homology detected between rodents, primates, and humans [13–15]. Foundational studies [10, 11] revealed that these two types of action control are controlled by distinct, parallel circuits in the cortico-striatal network (although more recent work has called into question how definitive these distinctions might be [16–18]). Classically, disrupting one circuit has been shown to shift behaviour to the other action control system, reflecting the behavioural changes seen in compulsive disorders. Despite these studies accurately reproducing aspects of behaviour change, however, the experimental approaches they employ (typically lesions or pharmacological inactivation) do not adequately model the subtle neural disturbances observed in disorders such as SUD and OCD, where widespread neuronal silencing or death is either absent, or present only late in disease progression, long after symptoms appear [3, 19, 20]. Therefore, the question remains as to what drives this shift in action control. Recent research implicates stress as a common precipitating factor in psychiatric disorders [18], and at a neural level, stress is almost certainly exerting this effect through neuroinflammation [21]. In accordance with this, striatal neuroinflammation has been consistently reported by post-mortem and neuroimaging studies of individuals with compulsive disorders [2, 4, 19, 22–25]. Accordingly, we modelled this physiologically relevant neuropathology in rats to determine the consequences for goal-directed versus habitual action control.

Specifically, we infused the gram-negative bacterial endotoxin and neuroinflammatory mimetic lipopolysaccharide (LPS) into the into the posterior dorsomedial striatum (pDMS) to induce a localised neuroinflammatory response [26–29]. We then assessed whether rats would show intact action selection across a range of assays probing both cue-guided and free operant choice behaviour. Among these, outcome devaluation procedures provided a particularly crucial test of goal-directed action, as intact performance (i.e., selective responding for a valued outcome over a devalued one) reflects both the sensitivity to outcome value and the contingency between the action and outcome: the two defining features of goal-direction [13, 30].

We targeted the pDMS in particular because of its established role as the ‘neuroanatomical locus of goal-directed action’ in rodents [13, 15], and its homology to the human caudate nucleus that expresses elevated neuroinflammatory markers in individuals with compulsive disorder [2–4, 22–25]. Behaviourally, striatal neuroinflammation produced a bias towards excessive goal-directed control and immunohistochemical results suggested a role for astrocytes in this behaviour. Thus, in a final experiment, we chemogeneticially activated hM4Di receptors expressed on pDMS astrocytes during the same behavioural assays to determine whether goal-directed action control depends on intact pDMS astrocytic function.

## Methods and materials

### Animals and housing conditions

A total of 176 Long-Evans rats, approximately half male, half females, weighing 180–350 g and 8–10 weeks of age at the beginning of each experiment were used for this study. Rats were purchased from the Australian Research Centre, Perth, Australia, and housed in groups of 2-3 in transparent amber plastic boxes located in a temperature- and humidity-controlled room with a 12-h light/dark (07:00–19:00 h light) schedule. During behavioural training and testing, animals were food restricted at ~85–95% (8–14 g chow per day, see supplement for full details). All procedures were approved by the Ethics Committees of the Garvan Institute of Medical Research, Sydney (AEC 18.34), Faculty of Science, University of Technology Sydney (ETH21-6657), and the University of Newcastle (A-2020-018).

### Surgery

For neuroinflammation experiments, stereotaxic surgery was performed to infuse LPS (5 µg/µL) into the pDMS (anteroposterior, −0.2 mm; mediolateral, ±2.4 mm (male), ±2.3 mm (female); and dorsoventral, −4.5 mm, relative to bregma) and another cohort of animals received LPS injected into their nucleus accumbens core (NAc core) (anteroposterior, 1.4 mm; mediolateral, ±2.2 mm; and dorsoventral, −7.5 mm, relative to bregma). For chemogenetic experiments, animals received bilateral injections of 1 µl per hemisphere of AAV-GFAP-hM4Di-mCherry (*Addgene*, item ID 50479-AAV5, titre 7 × 10¹² vg/mL) or the control AAV-GFAP104-mCherry (*Addgene*, item ID 58909-AAV5, titre 1 × 10¹³ vg/mL) at the coordinates for pDMS.

### Behavioural procedures

**Fig. 1:**
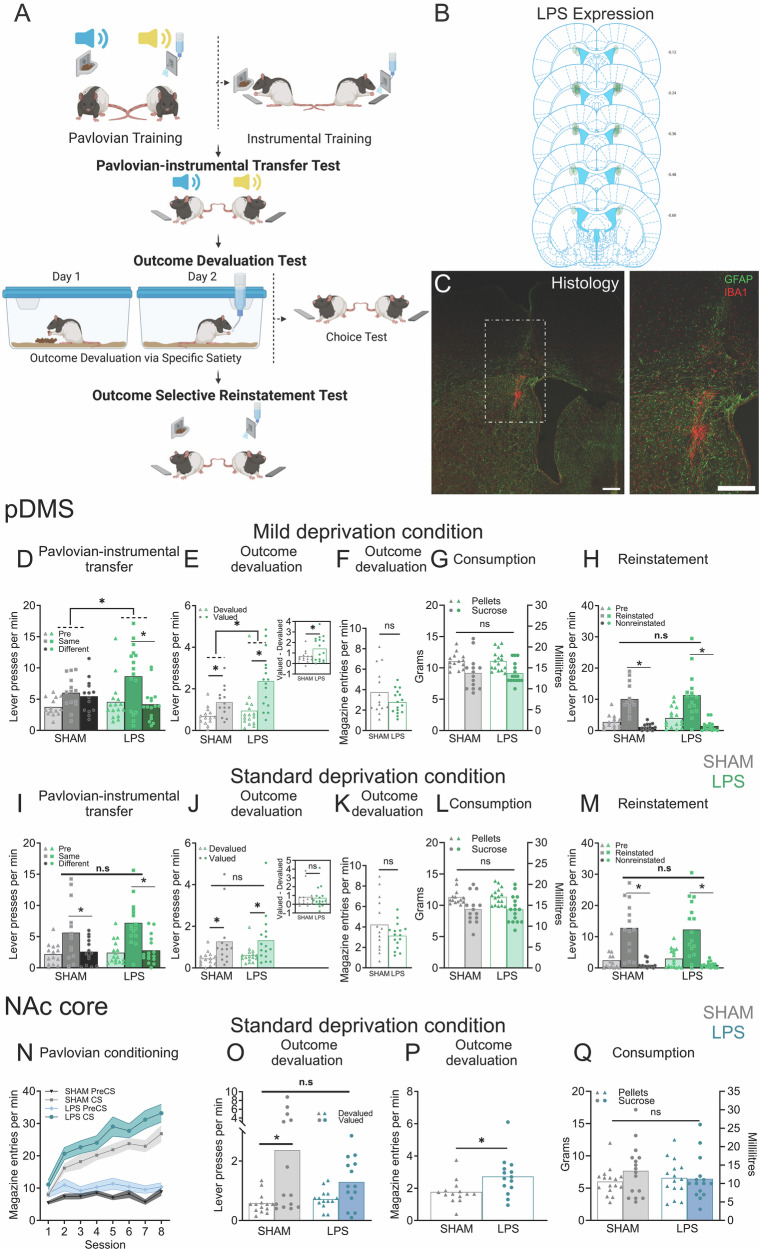
Striatal neuroinflammation causes excessive goal-directed action control in a region specific manner. **A** Experimental procedures for Pavlovian-instrumental transfer, outcome devaluation, and outcome-selective reinstatement, created with *Biorender*. **B** Distribution and locations of the lipopolysaccharide (LPS) injections in the posterior dorsomedial striatum (pDMS) included in the analysis. **C** pDMS image showing LPS placement as labelled with GFAP (glial fibrillary protein) and IBA1 (ionised calcium binding adaptor molecule 1) scale bars = 500 µm, (**D**–**H**) Individual data plots and (**D**, **E**, **H**) mean lever presses (**F**) magazine entries, or (**G**) grams/mL consumed during the (**D**) Pavlovian-instrumental transfer test, (**E**–**F**) outcome devaluation test, (**G**) pre-test feeding and (**H**) outcome-selective reinstatement test under mild deprivation conditions following pDMS LPS injections, (**I**–**M**) Individual data plots and (**I**, **J**, & **M**) mean lever presses, (**K**) magazine entries, or (**L**) grams/mL consumed during the (**I**) Pavlovian-instrumental transfer test, (**J**–**K**) outcome devaluation test, (**L**) consumption, and (**M**) outcome-selective reinstatement test under standard deprivation conditions following pDMS LPS injections. **N** Magazine entries per min (±SEM) during Pavlovian conditioning, (**O**) Individual data plots and mean lever presses during the outcome devaluation test, (**P**) data plots and mean magazine entries during the outcome devaluation test, and (**Q**) data plots and grams/mL consumed during pre-test feeding following NAc core LPS injections. *Denotes *p* < 0.05. (pDMS: *n* = 14 (SHAM), *n* = 16 (LPS), *N* = 30; NAc core: *n* = 14 (SHAM), *n* = 14 (LPS), *N* = 28).

**Fig. 2:**
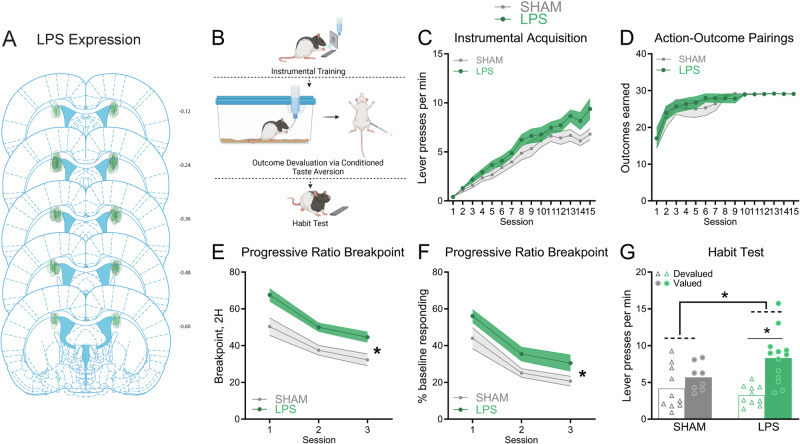
pDMS neuroinflammation prevents the formation of habits. **A** Distribution and localisation of lipopolysaccharide (LPS) injections within the posterior dorsomedial striatum (pDMS) included in the analysis. **B** Outcome devaluation procedure designed to promote habits, created with Biorender. **C** Lever pressing per min (±SEM), and (**D**) Number of action-outcome pairings (±SEM), during instrumental conditioning, (**E**) Breakpoint ( ± SEM) obtained during the 2-h, 3-day Progressive Ratio testing schedule, (**F**) Lever presses during progressive ratio testing (±SEM) presented as a percentage of baseline responding, (**G**) Individual data plots and mean lever presses during the outcome devaluation habit test. *Denotes *p* < 0.05. (*n* = 18 (SHAM), *n* = 23 (LPS), *N* = 41).

Behavioural procedures are described here and shown in Figs. 1A and 2A. For full details, please refer to the supplement.

### Pavlovian training

For experiments that involved Pavlovian training, rats were trained once per day for 8 days during which they received eight 2 min presentations of white noise or clicker (4 each) paired with either sucrose solution or pellet delivery.

### Lever press training

For the initial LPS and the final chemogenetic experiment, rats were trained over 8 days to press left and right levers for sucrose and grain pellets. Lever presses were initially continually reinforced, then progressed to a random ratio schedule. For the experiment employing experimental parameters intended to produce habits, rats received 8 days of two sessions per day, during which a single lever was pressed for sucrose, initially on continuous reinforcement, then on random interval schedules.

### Pavlovian instrumental transfer test

Each auditory cue (white noise and clicker) was presented four times (8 total) with levers continuously available but no outcomes delivered.

### Outcome devaluation

For the initial neuroinflammation and the final chemogenetic experiment, outcome devaluation was achieved using specific satiety. For the habit experiment, devaluation was achieved using conditioned taste aversion training. Tests were conducted with either one or both levers present and no outcomes delivered.

### Progressive ratio test

This test was administered during the habit experiment. Animals initially received a sucrose reward for a single lever press, then for 5 lever presses, then *n* + 5 lever presses until breakpoint—with breakpoint defined as 5 min of no lever pressing.

### Electrophysiology

Patch-clamp electrophysiology was performed on DMS tissue 4–6 weeks after local injections with either LPS or adeno-associated viruses (AAVs) resulting in transection with hM4Di-designer receptors exclusively activated by designer drugs (DREADDs). Putative medium spiny neuron (MSN) cell selection was based on MSN cell morphology and post-hoc confirmation of MSN delayed firing action potential (AP) profile, excluding cells without this profile from analysis. Full details are in the supplement.

### Imaging and immunofluorescence analysis

For quantification of glial fibrillary acidic protein (GFAP), ionised calcium binding adaptor molecule 1 (IBA1), and neuron specific nuclear protein (NeuN), a single image was taken of the pDMS and NAc core per hemisphere of each slice (6–10 images in total per brain region of each rat) on a Nikon TiE2 microscope using a 10x objective and Leica STELLARIS 20x air objective for representative images.

### Data and statistical analysis

Data were collected automatically by Med-PC and uploaded to Microsoft Excel using Med-PC to Excel software. Training data was analysed using two-way repeated measures ANOVAs, controlling the per-family error rate at *α* = 0.05. Test data were analysed using complex orthogonal contrasts controlling the per-contrast error rate at *α* = 0.05 according to the procedure described by Hays [31]. If interactions were detected, follow-up simple effects analyses (*α* = 0.05) were calculated to determine the source of the interaction.

## Results

### Dorsomedial striatal neuroinflammation produced excessive action control in rats

LPS placements in the pDMS are shown in Fig. 1B, C. Because we recently showed that neuroinflammation in the hippocampus of mice accelerated this region’s typical function in learning goal-directed actions [26] we suspected that neuroinflammation in pDMS might similarly enhance pDMS function to produce ‘excessive’ goal-directed action control, defined as animals exerting such control under conditions for which it is normally absent. Therefore, for the first series of experiments we created conditions to impair action selection in Sham animals by feeding rats a laboratory chow that is relatively high in fat and protein (see supplemental methods and Table 1 for details) on a mild deprivation schedule (approximately 90–95% of their initial body weight) to induce low levels of hunger and arousal [32].

We first tested cue-guided action selection using specific Pavlovian-instrumental transfer (Fig. 1A). Due to the mild deprivation conditions, we expected transfer to be impaired in controls, as evidenced by equal pressing on each lever regardless of the stimulus presented. However, we expected action selection to be intact in LPS rats despite these conditions, such that they would press more on the lever associated with the outcome predicted by the current stimulus (i.e., the pellet CS would elicit presses on the pellet lever, and likewise for sucrose, Same > Different). This prediction was confirmed (Fig. 1D). Entries into the food magazine and lever press responses did not differ between Sham and LPS groups during any phase of acquisition (largest *F* (1,28) = 1.085, *p* = 0.362, Supp. Figure 1A–C). On test there was no main effect of group, *F* < 1, but there was a group x transfer interaction, *F* (1,28) = 5.710, *p* = 0.024, driven by a significant simple effect of transfer (Same > Different) for the LPS group, *F* (1,28) = 15.996, *p* < 0.001, but not controls (Same = Different), *F* < 1.

Next, we assessed goal-directed control in the absence of stimuli using outcome devaluation (Fig. 1A). Given the mild deprivation conditions, we predicted that devaluation would be attenuated in Sham controls relative to the LPS group. As predicted, a group x devaluation interaction was observed, *F* (1,28) = 4.878, *p* = 0.035, with significant simple effect for both groups that was smaller for group Sham, *F* (1,28) = 7.445, *p* = 0.011, and larger for group LPS, *F* (1,28) = 31.060, *p* < 0.001 (Fig. 1E, inset shows data represented as a difference score [Valued – Devalued]). For this test, there was also a main effect of group, *F* (1,28) = 4.303, *p* = 0.047, indicating that group LPS responding more overall. Group differences were specific to lever pressing because groups did not differ in prefeeding consumption, *F* < 1 (Fig. 1G) or magazine entries during test, *F* < 1 (Fig. 1F).

Following instrumental retraining, rats were tested for outcome-selective reinstatement (Fig. 1A). Because selective reinstatement is not goal-directed [33], we expected it to remain unaffected by mild deprivation conditions or pDMS neuroinflammation. This was confirmed, as there was no main effect of group, *F* < 1, and both groups showed intact reinstatement, i.e., unexpected pellet delivery reinstated responding on the pellet lever, and sucrose delivery likewise reinstated responding on the sucrose lever (Reinstated > Nonreinstated, Fig. 1H), with a main effect of reinstatement, *F* (1,28) = 67.951, *p* < 0.001, that did not interact with group, *F* < 1.

Following this, we explored whether group differences persisted under standard deprivation conditions, [34–37] (see supplemental methods for details) for which goal-directed actions should be intact in sham controls and no longer ‘excessive’ in LPS animals. After brief retraining, rats underwent the same transfer, devaluation, and reinstatement tests. This time, performance did not differ between groups on any test: transfer main effect (Same > Different), *F* (1,28) = 30.605, *p* < 0.001 (Fig. 1I), devaluation main effect (Valued > Devalued), *F* (1,28) = 12.378, *p* < 0.001 (Fig. 1J, inset shows data as a difference score [Valued-Devalued]), reinstatement main effect (Reinstated > Nonreinstated), *F* (1,28) = 57.780, *p* < 0.001 (Fig. 1M), with no significant group main effects or interactions, all *F*s < 1.

### Neuroinflammation in ventromedial striatum (nucleus accumbens core) preserved instrumental responding, but increased sensitivity to Pavlovian food cues

To test the regional-specificity of neuroinflammation’s effect on goal-directed control, we injected LPS into the NAc core and repeated the same behavioural procedures [38, 39]. Training and testing were conducted under standard deprivation conditions, based on findings from a pilot study, which indicated that NAc core neuroinflammation was unlikely to produce excessive goal-directed control. LPS in the NAc core did not affect instrumental responding during either training or test (Supplementary Fig. 1G). Although NAc core neuroinflammation did appear to attenuate devaluation (Fig. 1O), this was not statistically supported as there was no group × lever interaction, *F* (1,25) = 2.858, *p* = 0.103. Rather, this attenuation likely resulted from a significant elevation in the competing magazine entry response, group main effect, *F* (1,26) = 6.02, *p* = 0.021 (Fig. 1P). LPS rats also made more food magazine entries during Pavlovian conditioning, main effect of group, *F* (1,25) = 6.962, *p* = 0.014 (Fig. 1N). Again, these differences were not due to changes in feeding or appetite, because prefeeding consumption did not differ between groups (*F* < 1, Fig. 1Q).

These results suggest that although NAc core neuroinflammation did not alter instrumental responding, it did enhance Pavlovian responding for food (magazine entries) when response competition from lever pressing was absent (i.e., Pavlovian training) or reduced due to satiety (i.e., devaluation testing). Because enhanced responding to Pavlovian cues has also been claimed to contribute to compulsive-like tendencies [40, 41], if translatable, these results suggest that differential distributions of neuroinflammation throughout the striatum could be a multifaceted source of compulsivity.

### Dorsomedial striatal neuroinflammation prevents rats from developing habits

We next wished to confirm that pDMS neuroinflammation could produce excessive goal-directed control under standard deprivation conditions by preventing habits. We trained a naïve cohort of rats on a single lever using a random interval schedule, as this has been reliably shown to produce habits [42, 43]; followed by a progressive ratio test to determine whether pDMS neuroinflammation had altered motivation per se (Fig. 2A). This was followed by devaluation by lithium chloride injections to induce conditioned taste aversion (Devalued group) whereas the Valued groups received injections of saline. Sham controls were expected to show habitual behaviour (Valued = Devalued), whereas, group LPS would remain goal-directed (Valued > Devalued).

Groups did not differ on lever press acquisition, though there was a trend toward greater responding in group LPS (Fig. 2B): main effect, *F* (1,39) = 3.36, *p* = 0.074. Importantly, the number of action-outcome pairings did not differ between groups, *F* < 1 (Fig. 2C). LPS rats did have increased breakpoints relative to Shams on progressive ratio testing, however, as there was a main effect of group, *F* (1,39) = 15.15, *p* < 0.0014 (Fig. 2D) that remained significant after correcting for baseline press rates, *F* (1,39) = 6.243, *p* = 0.0168 (Fig. 2E). As expected, during devaluation testing performance was sensitive to devaluation for LPS rats but not for controls (Fig. 2F). There was no main effect of group, *F* < 1, but there was a group × devaluation interaction, *F* (1,37) = 4.373, *p* = 0.043, comprised of intact devaluation in the LPS group (Valued > Devalued), *F* (1,37) = 20.198, *p* < 0.001, and not Shams (Valued = Devalued), *F* (1,37) = 1.417, *p* = 0.241. These findings suggest that pDMS neuroinflammation both increased motivation and sustained goal-directed control when controls were habitual.

### Immunohistochemical results indicate a role for astrocytes in excessive goal-directed control

**Fig. 3:**
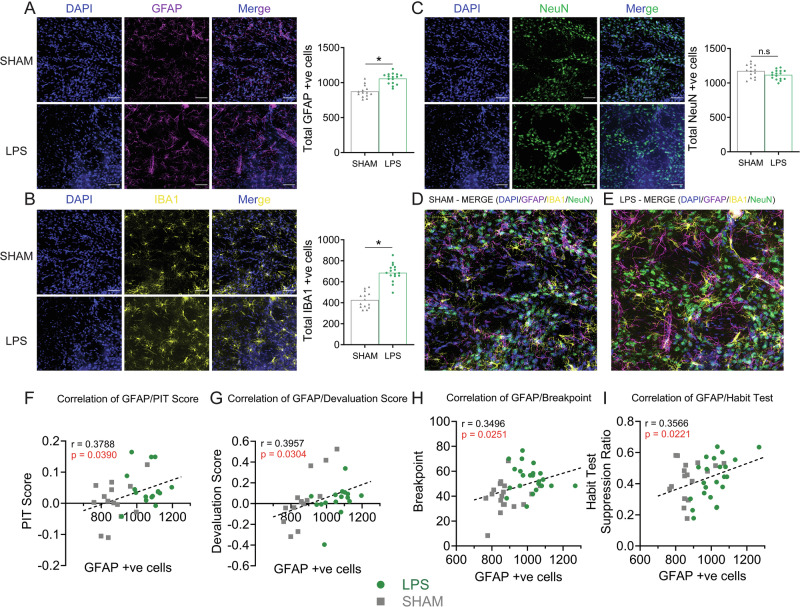
Injections of lipopolysaccharide (LPS) into posterior dorsomedial striatal (pDMS) increased the counts of GFAP and IBA1. Number of GFAP+ve cells positively correlated with excessive action control. Representative images of pDMS from a Sham (top panel) and LPS-injected rat (bottom panel) immunostained for DAPI and (**A**) GFAP, (**B**) IBA1, (**C**) NeuN, final graphs show individual data points and mean values for quantification of each, (**D**–**E**) Representative images of pDMS immunostained DAPI/GFAP/IBA1/NeuN merged from a Sham (**D**) and LPS-injected (**E**) rat, (**F**–**I**) Correlations between GFAP and behavioural performances. *Denotes *p* < 0.05, scale bars = 42 µm.

Our final aim was to investigate how pDMS neuroinflammation might cause excessive goal-directed control. To answer this, we first turned to immunohistochemical analyses of tissue from animals who underwent behavioural testing in Figs. 1 and 2 (Figs. 1B and 2B show the regions assessed). For pDMS animals in Fig. 1, rats in the LPS group showed significantly higher counts of cells positive for the astrocytic marker GFAP compared to Sham controls, *t* (28) = 6.26, *p* < 0.001 (Fig. 3A), and cells positive for the microglial marker IBA1, *t* (28) = 8.74, *p* < 0.001 (Fig. 3B), but no significant difference in NeuN-positive cells, a marker of neurons, *t* (28) = 1.90, *p* = 0.068 (Fig. 3C). A similar pattern of results was observed in tissue taken from animals in the habit formation experiment (Fig. 2), as shown in Supplementary Fig. 3B.

In addition to cell counts, rats in the LPS group also showed elevated signal intensities and other morphological changes in both the astrocyte marker GFAP and microglial marker IBA1 compared to controls (see Supp. Figure 3 for full results). However, with exception of breakpoint responding that correlated with IBA1 cell counts (Fig. S3C, bottom left), only GFAP measures significantly correlated with action selection on tests where group performances differed (Fig. 3F–I). Importantly, all correlations were calculated using lever press rates normalised to baseline responding, ensuring that observed associations reflect selectivity in behaviour (e.g., Valued > Devalued) rather than general increases in responding. Therefore, while both astrocytic and microglial proliferation were associated with the increase in motivation, only astrocytic proliferation was associated with the enhanced selectivity of actions.

### Neuroinflammation and chemogenetic excitation of Gi-coupled receptors on astrocytes differentially altered the firing properties of adjacent medium spiny neurons

If altered astrocytic functioning underlies the changes in goal-directed actions, it likely does so by altering the activity of nearby neurons, because astrocytes do not have long enough processes to interact with the broader neural circuit of goal-directed control. To explore this, we used in vitro whole cell patch clamp electrophysiology to determine how LPS injections in pDMS altered the firing properties of medium spiny neurons (MSNs).

**Fig. 4:**
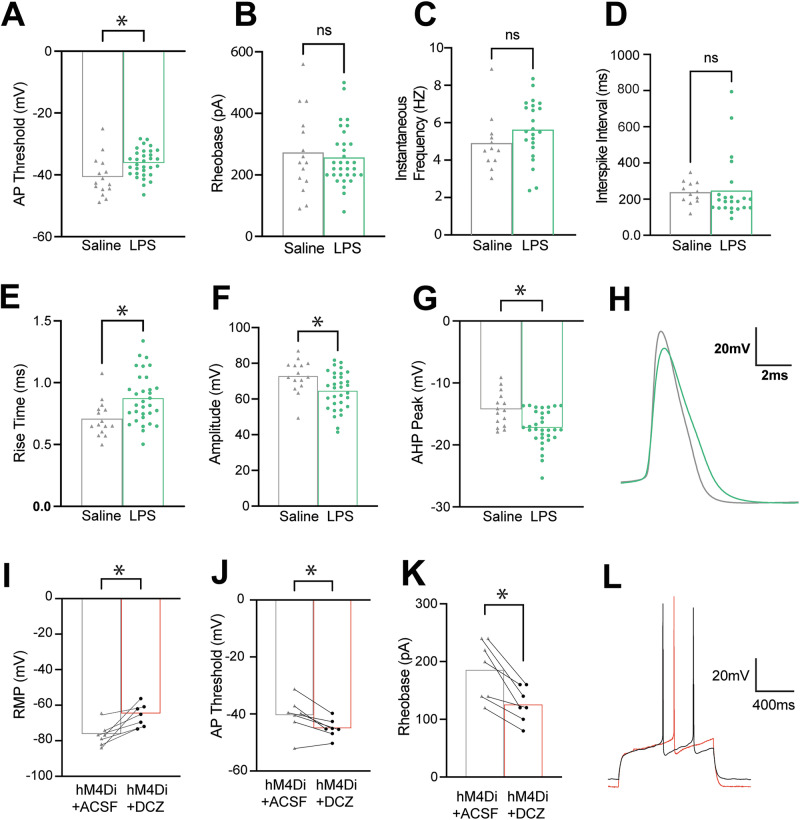
Electrophysiological changes to medium spiny neuron (MSN) action potential (AP) profile and discharge characteristics in pDMS with neuroinflammation or following chemogenetic activation of the Gi-pathway in astrocytes. **A**–**H** Results of whole-cell patch clamp electrophysiology recordings from MSNs following LPS or sham injections into the pDMS. **A**–**D** Individual data points showing AP threshold for each MSN voltage clamped at −80 mV, (**B**) rheobase, (**C**) instantaneous frequency, or (**D**) interspike interval for each MSN. Individual data points for changes to AP profile for each MSN voltage clamped at −80 mV, including (**E**) AP rise time, (**F**) AP amplitude, and (**G**) an afterhyperpolarisation (AHP) peak. **H** Example cell average trace shows the AP profile characteristics of rise time and amplitude (LPS = green, saline = grey). **I**–**L** Results of whole-cell patch clamp electrophysiology recordings from MSNs following the application of artificial cerebrospinal fluid (ACSF), then designer receptors exclusively activated by designer drugs (DREADD) agonist deschloroclozapine (DCZ) to astrocytes transfected with hM4Di DREADDs. Individual data points showing (**I**) resting membrane potential (RMP), (**J**) AP threshold, and (**K**) rheobase for each MSN. **L** Example cell rheobase traces (ASCF = grey, DCZ = orange). LPS vs saline; LPS at RMP *n* = 33 cells and at −80 voltage clamp *n* = 32 cells, from *n* = 4 animals; saline; *n* = 15 cells from *n* = 3 animals. GFAP-HM4Di *n* = 7 cells from *n* = 2 animals tested with ACSF then DCZ.

We bilaterally injected LPS or saline into the pDMS of rats, then recorded from acute brain slices 6 weeks later. Recordings were first taken at resting membrane potential (RMP, Fig. S4), then repeated while cells were voltage-clamped at -80mV, consistent with the reported in vivo RMP of MSNs [44]. LPS MSNs displayed a more depolarised AP threshold following a depolarising current steps protocol when voltage-clamped at −80 mV (t20.49 = 2.46, *p* = 0.023, Fig. 4A), whereas no changes were seen for rheobase, instantaneous frequency, or interspike interval (Fig. 4B–D). The first AP also showed increased rise time (t37.34 = 3.21, *p* = 0.003, Fig. 4E, H) and decreased amplitude (t31.31 = 2.72, *p* = 0.011, Fig. 4F, H) in LPS MSNs. Furthermore, LPS MSNs showed a significantly more depolarised afterhyperpolarization (AHP) peak (t28.37 = 3.40, *p* = 0.002, Fig. 4G, H). No changes were seen in latency to first spike, half-width, or AHP position (data not shown). These firing patterns suggested that LPS-affected cells in pDMS were less likely to be activated than controls. Taken in conjunction with behavioural results, these findings suggest that LPS in pDMS disrupts the precise excitatory/inhibitory balance necessary for appropriate control over actions, causing goal-directed control to be excessive.

We next manipulated astrocytes specifically. Consistent with the increase in GFAP expression (Fig. 3) a previous study that chemogenetically activated hM3Dq DREADDs on DMS astrocytes observed excessive goal-directed control, albeit in mice undergoing different behavioural procedures [45]. We therefore aimed to extend these findings by employing a procedure that would reveal how pDMS astrocytes contribute to goal-directed control in their homoeostatic form. Based on evidence that Gi-G-protein-coupled receptors (GPCR) are highly expressed on striatal astrocytes [46], and findings that activating these receptors has been shown to ‘correct’ a number of Huntington-like [47] and compulsion-like [48] deficits in mice, we used astrocyte-specific hM4Di DREADDs to examine the consequences of Gi pathway activation on neuronal firing and action selection.

Although prior studies have investigated the activation of astrocytic Gi-GPCRs in the striatum [46, 47], they have primarily focused on the dorsolateral rather than dorsomedial compartment. This is important because there are now several studies demonstrating the regional specificity of astrocyte function within the brain, even within the striatum [46, 47, 49, 50]. Thus, to establish the effects of astrocytic hM4Di activation on neuronal firing properties, we bilaterally injected GFAP-hM4Di-DREADD into the pDMS, and recorded from 7 cells across two animals, firstly in artificial cerebrospinal fluid (ACSF) and then following bath application of hM4Di-DREADD agonist DCZ (1 µM). After DCZ application, RMP was significantly more depolarised (t6.00 = 4.14, *p* = 0.0018, Fig. 4I), shifting cells closer to AP threshold. Then, following the same depolarising current steps protocol used in LPS electrophysiology experiments, AP threshold was lower at RMP (t6.00 = 3.57, *p* = 0.012, Fig. 4J), further narrowing the range between RMP and AP threshold. Rheobase was also significantly reduced following DCZ application (t6.00 = 4.86, *p* = 0.003 Fig. 4K, L). No other changes were seen in AP profile or firing properties (Supplementary Fig. 4F–K) with DCZ application at RMP. Recordings were also taken while cells were voltage-clamped at −80 mV (Supplementary Table 5), blocking the depolarisation of RMP induced by DCZ, and this resulted in no changes to AP profile or firing properties.

This profile of MSN firing contrasts with that produced by LPS injection, and to the results of Kang et al. [45], who found that activation of hM3Dq-transfected astrocytes reduced both excitatory and inhibitory postsynaptic potentials (EPSPs and IPSPs) in MSNs. Given that both LPS and hM3Dq activation in astrocytes facilitated goal-directed control whilst producing a distinct profile of neuronal firing, we hypothesised that the activation of Gi receptors on pDMS astrocytes would abolish goal-directed control. Although this may seem counterintuitive in light of prior findings that lesioning or inactivating this structure also abolishes action control [51, 52], recent findings paint a more nuanced picture of the conditions necessary for goal-directed action [53, 54]. In particular, these studies propose that spatially organised neuronal ensembles within the striatum must behave in a precise and complementary manner (referred to as “behavioural syllables”) to produce accurate action selection [46]. Our electrophysiology results suggest that activating the Gi pathway in pDMS astrocytes disrupts this precision, which we expect to disrupt the behavioural selectivity necessary for goal-directed control.

### Chemogenetic activation of the Gi-pathway in dorsomedial striatal astrocytes abolished goal-directed action control

**Fig. 5:**
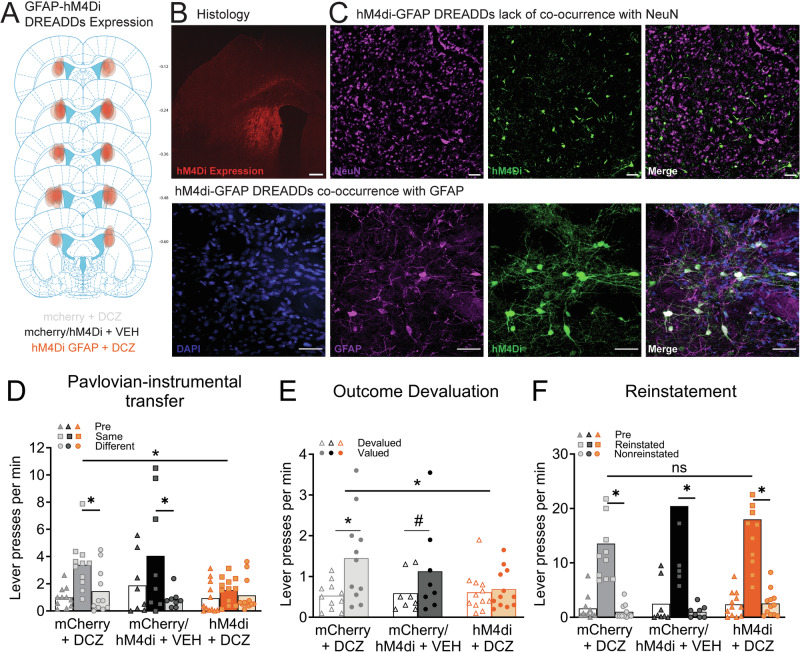
Chemogenetic activation of the Gi-pathway in pDMS astrocytes abolished goal-directed action control. **A** Diagrammatic representation of the distribution and locations of the viral expressions in the pDMS included in the analysis. **B** Histological verification of the GFAP virus expression in pDMS (scale bar = 500 µm). **C** Representative images showing lack of colocalization with NeuN (top panel) and colocalization of mCherry from GFAP-hM4D-Gi-DREADD virus with the GFAP (bottom panel) scale bars = 45 µm. Individual data plots and mean lever presses during the (**D**) Pavlovian-instrumental transfer test, (**E**) outcome devaluation test, and (**F**) outcome-selective reinstatement test. *Denotes that the *p* < 0.05, #denotes *p* = 0.055. (*n* = 8 (hM4Di/mCherry + VEH [hM4Di+Veh = 5 + mCherry+Veh *n* = 3]), *n* = 11 (mCherry + DCZ), *n* = 12 (hM4Di + DCZ), *N* = 31).

Above results suggest that the change in astrocyte activity and morphology that occurs as part of the neuroinflammatory response leads to an excessive reliance on goal-directed actions. This implies that the intact signalling of astrocytes in their homoeostatic form—i.e., astrocytes that have not undergone a phenotypic shift to a pro-inflammatory-like state—is necessary for intact goal-directed control. To test this idea, behavioural experiments employing chemogenetics were conducted under standard deprivation conditions. Figure 5A and the bottom left panel of Fig. 5B show the representative placements of AAV transfection in the pDMS. The bottom panel of Fig. 5C shows extensive co-localisation of GFAP and AAV-hM4Di-GFAP-mCherry, and the top panel shows lack of overlap with NeuN, confirming the specificity of transfection for astrocytes.

Pavlovian and instrumental training were conducted without DCZ administration and proceeded without incident (Supplementary Fig. 5A–C, all group Fs < 1). However, animals did receive vehicle or DCZ injections to activate the astrocytic Gi pathway 25–30 min prior to each test. This prevented transfer, which was impaired (Same =  Different) in animals that received both the active virus and DCZ (hM4Di+DCZ) but intact (Same > Different) for both vehicle (mCherry or hM4Di+Veh) and DCZ-only (mCherry+DCZ) controls (Fig. 5D). There was a marginal reduction in overall responding, hM4Di+DCZ vs. controls comparison, *F* (1,28) = 3.96, *p* = 0.056 (comparison between controls, *F* < 1). More importantly, a group × transfer interaction, *F* (1,28) = 4.947, *p* = 0.034, consisting of intact simple effects for groups mCherry+DCZ, *F* (1,28) = 5.995, *p* = 0.021, and hM4Di+Veh, *F* (1,28) = 11.731, *p* = 0.002, but not group hM4Di + DCZ, *F* < 1.

This also prevented outcome devaluation, which was intact (Valued > Devalued) for controls but abolished (Valued = Devalued) for group hM4Di+DCZ (Fig. 5D). Specifically, there were no differences in overall responding (both group main effect *F*s < 1), but there was a group × devaluation interaction, *F* (1,28) = 5.494, *p* = 0.026, driven by intact devaluation simple effects in group hM4Di + Veh, *F* (1,28) = 16.464, *p* < 0.001, a marginal simple effect in mCherry + DCZ, *F* (1,28) = 4.063, *p* = 0.054, but no effect for group hM4Di + DCZ *F* < 1.

Finally, performance on selective reinstatement was again intact for all groups; there were no group main effects (largest *F* was for the comparison between the control groups, *F* (1,28) = 1.726, *p* = 0.2), but there was a reinstatement main effect (Reinstated > Nonreinstated) *F* (1,28) = 67.965, *p* < 0.001, that did not interact with group *F* < 1 (Fig. 5E). This demonstrates that the activation of the Gi pathway in pDMS astrocytes does not simply replicate the behavioural results observed following a pDMS lesion or inactivation which have been shown to abolish reinstatement [51] as well as devaluation [51, 52] and transfer [55]. Rather, these findings demonstrate a distinct role for astrocytes in regulating the neuronal activity necessary for goal-directed control.

## Discussion

Here, we show that striatal neuroinflammation, a common neuropathological feature of compulsive disorders, drives excessive goal-directed action in rats. First, LPS-induced pDMS neuroinflammation promoted such actions under conditions that typically elicit habits. These effects were behaviourally specific, as they did not alter food consumption or selective reinstatement. They were also anatomically specific: NAc core neuroinflammation did not significantly disrupt instrumental responding. Electrophysiological recordings revealed that, overall, pDMS neuroinflammation reduced the propensity of MSNs to fire, whereas the chemogenetic activation of hM4Di receptors on astrocytes increased MSN firing tendencies. Consistent with these conflicting effects, in vivo astrocytic Gi activation disrupted rather than promoted goal-directed actions.

These findings support the emerging hypothesis that individuals with striatal neuroinflammation, such as those with compulsive disorders, are acting with cognitive control, albeit inappropriately, under conditions that would otherwise elicit habits [9, 56]. A potential confound to this interpretation is the observation that rats with striatal neuroinflammation also exhibited higher breakpoints on the progressive ratio test, indicating higher levels of motivation. This could, in principle, explain why the LPS group in the earlier cohort displayed goal-directed control under conditions of low deprivation. However, this account cannot explain why LPS rats also remained goal-directed when Sham rats trained under standard deprivation conditions had transitioned to habitual control (i.e., Fig. 2F). Indeed, increased motivation is typically associated with faster habit formation rather than resistance to it [57, 58]. Thus, while the elevated motivation likely contributed to some aspects of performance, it cannot account for the full series of behavioural results. Nevertheless, these findings underscore the complex and sometimes counterintuitive interplay between motivation, habit formation, and goal-directed control.

Although our primary focus was on elucidating neural mechanisms of decision-making, current findings may also offer translational insight into behaviours observed across psychiatric conditions where action control is disrupted. For example, the interpretation of ‘excessive goal-direction’ fits a range of clinical phenomena, including the extreme lengths individuals with SUD undertake to obtain drugs [59], or the momentary feeling of relief experienced by individuals with OCD after performing compulsive actions [60]. It further aligns with observations that individuals with Parkinson’s disease (who also exhibit significant striatal neuroinflammation [61]) are overly goal-directed, which can slow their responses [62]. Current findings regarding cue-guided action selection are similarly consistent with enhanced Pavlovian-instrumental transfer effects in rats that have learned to self-administer methamphetamine [63], and in humans with alcohol use disorder [64–66].

Importantly, we do not claim to model OCD or SUD directly. Rather, we interpret our results as identifying a potential mechanism (striatal neuroinflammation) that may influence decision-making strategies relevant to, but not diagnostic of, these and similar disorders (e.g., Paediatric Autoimmune Neuropsychiatric Disorders Associated with Streptococcal Infections [PANDAS]). If our findings do translate, however, it does bring into question why several lines of evidence suggest that individuals with OCD and SUD over-rely on habits [5–7]. The following points help reconcile these views. First, individuals with compulsive disorders often show intense focus on a single goal while neglecting competing ones. Thus, if the studies linking compulsivity to habits used goals that weren’t personally salient, participants may have been unmotivated or unable to direct their actions towards them. Second, neuroinflammation is unevenly distributed throughout the brains of individuals with compulsive disorders [1] and, as seen here and in past work [26, 67], neuroinflammation in different brain regions produces distinct behavioural outcomes. An individual’s dominant behavioural strategy could therefore depend on which brain regions are most affected or could fluctuate dependent on environmental conditions that might preferentially drive cortical, thalamic, and/or nigral/tegmental inputs to different regions. Finally, there is growing evidence that neuronal ensembles within the striatum encode specific sets of action-outcome contingencies [54, 68, 69]. Depending on the distribution of neuroinflammation and how this interacts with these ensembles, this could promote behaviour that is more goal-directed or more habitual, respectively.

Current results point to astrocytes interacting with these neuronal ensembles to produce goal-directed actions, and it is interesting to consider how this might be achieved. Goal-directed actions are defined by their specificity in achieving a particular goal, such as pressing a lever for a unique outcome (e.g., left lever→pellets, right lever→sucrose), whereas habits are elicited based on non-specific, prior experience of reinforcement [70]. A goal-directed response thus requires the selective activation of the neural ensemble that stores the correct response-outcome association, as well as inhibition of the alternate ensemble. Habitual responding does not, instead relying on procedural processes encoded by dorsolateral striatum [71]. For goal-directed actions, these ensembles consist of the precise, temporally coordinated firing of dopamine 1 (D1) and D2-expressing MSNs [53, 54, 72]. Astrocytes are well-positioned to modulate this activity as they contact both D1 and D2-expressing MSNs to a similar extent [73] and express D1 receptors that mediate the dopamine-evoked depression of excitatory neurotransmission [74].

Finally, it is worth acknowledging two limitations of our findings. First, although the LPS-induced neuroinflammation in this study had likely progressed past the acute phase by the time of behavioural testing (4–8 weeks post-surgery), it may not fully recapitulate the chronic neuroinflammatory profile experienced by individuals with long-term disorders. This raises the possibility that the behavioural impact of striatal neuroinflammation could shift over time as the neuroinflammatory response evolves [75]. Second, although current results support a role for homoeostatic astrocytic function in goal-directed action control, they do not exclude contributions from other mechanisms, such as the phenotypic responses of microglia. Future studies may wish to address these questions.

In summary, our findings indicate that the alterations to action control experienced by individuals with compulsive disorders are unlikely to be reduced to a single mechanism [11], but are multifactorial [76], and identify striatal astrocytes as a novel potential therapeutic target to restore adaptive action control. Future research should aim to clarify how different neural and glial mechanisms interact to shape decision-making strategies across contexts and over time.

## Supplementary information

Supplemental Material for manuscript

## Data Availability

10.17605/OSF.IO/297ZU.

## References

[1] Kohno M, Link J, Dennis LE, McCready H, Huckans M, Hoffman WF, et al. Neuroinflammation in addiction: a review of neuroimaging studies and potential immunotherapies. Pharmacol Biochem Behav. 2019;179:34–42. 30695700 10.1016/j.pbb.2019.01.007 PMC6637953

[2] Attwells S, Setiawan E, Wilson AA, Rusjan PM, Mizrahi R, Miler L, et al. Inflammation in the neurocircuitry of obsessive-compulsive disorder. JAMA Psychiatry. 2017;74:833–40. 28636705 10.1001/jamapsychiatry.2017.1567 PMC5710556

[3] De La Monte SM, Kril JJ. Human alcohol-related neuropathology. Acta Neuropathol. 2014;127:71–90. 24370929 10.1007/s00401-013-1233-3 PMC4532397

[4] Cadet JL, Bisagno V, Milroy CM. Neuropathology of substance use disorders. Acta Neuropathol. 2014;127:91–107. 24292887 10.1007/s00401-013-1221-7 PMC7453825

[5] Gillan CM, Robbins TW, Sahakian BJ, Van Den Heuvel OA, Van Wingen G. The role of habit in compulsivity. European Neuropsychopharmacol. 2016;26:828–40. 10.1016/j.euroneuro.2015.12.033 PMC4894125 26774661

[6] Everitt BJ, Robbins TW. Neural systems of reinforcement for drug addiction: from actions to habits to compulsion. Nat Neurosci. 2005;8:1481–9. 16251991 10.1038/nn1579

[7] Gillan CM, Morein-Zamir S, Urcelay GP, Sule A, Voon V, Apergis-Schoute AM, et al. Enhanced avoidance habits in obsessive-compulsive disorder. Biol Psychiatry. 2014;75:631–8. 23510580 10.1016/j.biopsych.2013.02.002 PMC3988923

[8] Lüscher C, Robbins TW, Everitt BJ. The transition to compulsion in addiction. Nat Rev Neurosci. 2020;21:247–63. 32231315 10.1038/s41583-020-0289-z PMC7610550

[9] Hogarth L. Addiction is driven by excessive goal-directed drug choice under negative affect: translational critique of habit and compulsion theory. Neuropsychopharmacol. 2020;45:720–35. 10.1038/s41386-020-0600-8 PMC7265389 31905368

[10] Epstein DH. Let’s agree to agree: a comment on Hogarth (2020), with a plea for not-so-competing theories of addiction. Neuropsychopharmacology. 2020;45:715–6. 31969695 10.1038/s41386-020-0618-y PMC7265294

[11] Vandaele Y, Ahmed SH. Habit, choice, and addiction. Neuropsychopharmacol. 2021;46:689–98. 10.1038/s41386-020-00899-y PMC8027414 33168946

[12] Berridge KC. Comment on vandaele and ahmed: rethinking habits in addiction. Neuropsychopharmacol. 2021;46:687–8. 10.1038/s41386-020-00932-0 PMC8027213 33323944

[13] Balleine BW. The meaning of behavior: discriminating reflex and volition in the brain. Neuron. 2019;104:47–62. 31600515 10.1016/j.neuron.2019.09.024

[14] Balleine BW, O’Doherty JP. Human and rodent homologies in action control: corticostriatal determinants of goal-directed and habitual action. Neuropsychopharmacol. 2010;35:48–69. 10.1038/npp.2009.131 PMC3055420 19776734

[15] Bradfield LA, Balleine BW. “The learning and motivational processes controlling goal-directed action and their neural bases” in Decision Neuroscience 71–80 (Elsevier, 2017; https://linkinghub.elsevier.com/retrieve/pii/B9780128053089000063).

[16] Seiler JL, Cosme CV, Sherathiya VN, Schaid MD, Bianco JM, Bridgemohan AS, et al. Dopamine signaling in the dorsomedial striatum promotes compulsive behavior. Current Biol. 2022;32:1175–1188.e5. 10.1016/j.cub.2022.01.055 PMC8930615 35134327

[17] Van Elzelingen W, Goedhoop J, Warnaar P, Denys D, Arbab T, Willuhn I. A unidirectional but not uniform striatal landscape of dopamine signaling for motivational stimuli. Proc Natl Acad Sci USA. 2022;119:e2117270119. 35594399 10.1073/pnas.2117270119 PMC9171911

[18] Giovanniello JR, Paredes N, Wiener A, Ramírez-Armenta K, Oragwam C, Uwadia HO, et al. A dual-pathway architecture for stress to disrupt agency and promote habit. Nature. 2025; 10.1038/s41586-024-08580-w. 10.1038/s41586-024-08580-w PMC12011321 39972126

[19] Maia TV, Cooney RE, Peterson BS. The neural bases of obsessive-compulsive disorder in children and adults. Dev Psychopathol. 2008;20:1251–83. 18838041 10.1017/S0954579408000606 PMC3079445

[20] Pando-Naude V, Toxto S, Fernandez-Lozano S, Parsons CE, Alcauter S, Garza-Villarreal EA. Gray and white matter morphology in substance use disorders: a neuroimaging systematic review and meta-analysis. Transl Psychiatry. 2021;11:29. 33431833 10.1038/s41398-020-01128-2 PMC7801701

[21] McEwen BS, Bowles NP, Gray JD, Hill MN, Hunter RG, Karatsoreos IN, et al. Mechanisms of stress in the brain. Nat Neurosci. 2015;18:1353–63. 26404710 10.1038/nn.4086 PMC4933289

[22] Mews P, Cunningham AM, Scarpa J, Ramakrishnan A, Hicks EM, Bolnick S, et al. Convergent abnormalities in striatal gene networks in human cocaine use disorder and mouse cocaine administration models. Sci Adv. 2023;9:eadd8946. 36763659 10.1126/sciadv.add8946 PMC9916993

[23] Saxena S, Rauch SL. Functional neuroimaging and the neuroanatomy of obsessive-compulsive disorder. Psychiatric Clin North Am. 2000;23:563–86. 10.1016/s0193-953x(05)70181-7 10986728

[24] Piantadosi SC, Manning EE, Chamberlain BL, Hyde J, LaPalombara Z, Bannon NM, et al. Hyperactivity of indirect pathway-projecting spiny projection neurons promotes compulsive behavior. Nat Commun. 2024;15:4434. 38789416 10.1038/s41467-024-48331-z PMC11126597

[25] Frick L, Pittenger C. Microglial dysregulation in OCD, tourette syndrome, and PANDAS. J Immunol Res. 2016;2016:1–8. 10.1155/2016/8606057 PMC5174185 28053994

[26] Ganesan K, Ghorbanpour S, Kendall W, Broome ST, Gladding JM, Dhungana A, et al. Hippocampal neuroinflammation induced by lipopolysaccharide causes sex-specific disruptions in action selection, food approach memories, and neuronal activation. Brain Behav Immun. 2025;124:9–27. 39547520 10.1016/j.bbi.2024.11.011

[27] Becchi S, Chieng B, Bradfield LA, Capellán R, Leung BK, Balleine BW. Cognitive effects of thalamostriatal degeneration are ameliorated by normalizing striatal cholinergic activity. Sci Adv. 2023;9:eade8247. 37352346 10.1126/sciadv.ade8247 PMC10289650

[28] Valenzuela-Arzeta IE, Soto-Rojas LO, Flores-Martinez YM, Delgado-Minjares KM, Gatica-Garcia B, Mascotte-Cruz JU, et al. LPS triggers acute neuroinflammation and Parkinsonism involving NLRP3 inflammasome pathway and mitochondrial CI dysfunction in the Rat. Int J Mol Sci. 2023;24:4628. 36902058 10.3390/ijms24054628 PMC10003606

[29] Na S, Duan X, Wang R, Fan Y, Xue K, Tian S, et al. Chronic neuroinflammation induced by lipopolysaccharide injection into the third ventricle induces behavioral changes. J Mol Neurosci. 2021;71:1306–19. 33405196 10.1007/s12031-020-01758-7

[30] Balleine BW, Dickinson A. Goal-directed instrumental action: contingency and incentive learning and their cortical substrates. Neuropharmacology. 1998;37:407–19. 9704982 10.1016/s0028-3908(98)00033-1

[31] Hays WL. Statistics for the social sciences. New York, Holt, Rinehart, & Winston, 1973.

[32] Becchi S, Hood J, Kendig MD, Mohammadkhani A, Shipman ML, Balleine BW, et al. Food for thought: diet-induced impairments to decision-making and amelioration by N-acetylcysteine in male rats. Psychopharmacology. 2022;239:3495–506. 36219247 10.1007/s00213-022-06223-4

[33] Ostlund SB, Balleine BW. Selective reinstatement of instrumental performance depends on the discriminative stimulus properties of the mediating outcome. Animal Learn Behav. 2007;35:43–52. 17557390 10.3758/bf03196073

[34] Ma C, Jean-Richard-dit-Bressel P, Roughley S, Vissel B, Balleine BW, Killcross S, et al. Medial orbitofrontal cortex regulates instrumental conditioned punishment, but not pavlovian conditioned fear. Cerebral Cortex Commun. 2020;1:tgaa039. 10.1093/texcom/tgaa039 PMC8152850 34296108

[35] Bradfield LA, Leung BK, Boldt S, Liang S, Balleine BW. Goal-directed actions transiently depend on dorsal hippocampus. Nat Neurosci. 2020;23:1194–7. 32778789 10.1038/s41593-020-0693-8

[36] Bradfield LA, Balleine BW. Thalamic control of dorsomedial striatum regulates internal state to guide goal-directed action selection. J Neurosci. 2017;37:3721–33. 28242795 10.1523/JNEUROSCI.3860-16.2017 PMC6596916

[37] Bradfield LA, Dezfouli A, van Holstein M, Chieng B, Balleine BW. Medial orbitofrontal cortex mediates outcome retrieval in partially observable task situations. Neuron. 2015;88:1268–80. 26627312 10.1016/j.neuron.2015.10.044

[38] Corbit LH, Muir JL, Balleine BW. The role of the nucleus accumbens in instrumental conditioning: evidence of a functional dissociation between accumbens core and shell. J Neurosci. 2001;21:3251–60. 11312310 10.1523/JNEUROSCI.21-09-03251.2001 PMC6762583

[39] Hart G, Bradfield LA, Fok SY, Chieng B, Balleine BW. The bilateral prefronto-striatal pathway is necessary for learning new goal-directed actions. Curr Biol. 2018;28:2218–2229.e7. 30056856 10.1016/j.cub.2018.05.028

[40] Robinson T, Berridge KC. The neural basis of drug craving: an incentive-sensitization theory of addiction. Brain Res Rev. 1993;18:247–91. 8401595 10.1016/0165-0173(93)90013-p

[41] Bradfield L, Morris R, Balleine BW. “Obsessive-compulsive disorder as a failure to integrate goal-directed and habitual action control” in Obsessive-compulsive disorder: phenomenology, pathophysiology, and treatment 343 (Oxford University Press, 2017).

[42] Adams CD, Dickinson A. Instrumental responding following reinforcer devaluation. Quarterly J Exp Psychol Sect B. 1981;33:109–21.

[43] Lingawi NW, Balleine BW. Amygdala central nucleus interacts with dorsolateral striatum to regulate the acquisition of habits. J Neurosci. 2012;32:1073–81. 22262905 10.1523/JNEUROSCI.4806-11.2012 PMC3711777

[44] Gertler TS, Chan CS, Surmeier DJ. Dichotomous anatomical properties of adult striatal medium spiny neurons. J Neurosci. 2008;28:10814–24. 18945889 10.1523/JNEUROSCI.2660-08.2008 PMC3235748

[45] Kang S, Hong S-I, Lee J, Peyton L, Baker M, Choi S, et al. Activation of astrocytes in the dorsomedial striatum facilitates transition from habitual to goal-directed reward-seeking behavior. Biol Psychiatry. 2020;88:797–808. 32564901 10.1016/j.biopsych.2020.04.023 PMC7584758

[46] Khakh BS. Astrocyte-neuron interactions in the striatum: insights on identity, form, and function. Trends Neurosci. 2019;42:617–30. 31351745 10.1016/j.tins.2019.06.003 PMC6741427

[47] Yu X, Nagai J, Marti-Solano M, Soto JS, Coppola G, Babu MM, et al. Context-specific striatal astrocyte molecular responses are phenotypically exploitable. Neuron. 2020;108:1146–62.e10. 33086039 10.1016/j.neuron.2020.09.021 PMC7813554

[48] Soto JS, Neupane C, Kaur M, Pandey V, Wohlschlegel JA, Khakh BS. Astrocyte Gi-GPCR signaling corrects compulsive-like grooming and anxiety-related behaviors in Sapap3 knockout mice. Neuron 2024;112:3412–23.e6. 10.1016/j.neuron.2024.07.019 PMC11512628 39163865

[49] Chai H, Diaz-Castro B, Shigetomi E, Monte E, Octeau JC, Yu X, et al. Neural circuit-specialized astrocytes: transcriptomic, proteomic, morphological, and functional evidence. Neuron. 2017;95:531–549.e9. 28712653 10.1016/j.neuron.2017.06.029 PMC5811312

[50] Huang AY-S, Woo J, Sardar D, Lozzi B, Bosquez Huerta NA, Lin C-CJ, et al. Region-specific transcriptional control of astrocyte function oversees local circuit activities. Neuron. 2020;106:992–1008.e9. 32320644 10.1016/j.neuron.2020.03.025 PMC7879989

[51] Yin HH, Ostlund SB, Knowlton BJ, Balleine BW. The role of the dorsomedial striatum in instrumental conditioning. Eur J Neurosci. 2005;22:513–23. 16045504 10.1111/j.1460-9568.2005.04218.x

[52] Yin HH, Knowlton BJ, Balleine BW. Blockade of NMDA receptors in the dorsomedial striatum prevents action-outcome learning in instrumental conditioning. Eur J Neurosci. 2005;22:505–12. 16045503 10.1111/j.1460-9568.2005.04219.x

[53] Peak J, Chieng B, Hart G, Balleine BW. Striatal direct and indirect pathway neurons differentially control the encoding and updating of goal-directed learning. eLife. 2020;9:e58544. 33215609 10.7554/eLife.58544 PMC7707820

[54] Matamales M, McGovern AE, Mi JD, Mazzone SB, Balleine BW, Bertran-Gonzalez J. Local D2- to D1-neuron transmodulation updates goal-directed learning in the striatum. Science. 2020;367:549–55. 32001651 10.1126/science.aaz5751

[55] Corbit LH, Janak PH. Posterior dorsomedial striatum is critical for both selective instrumental and Pavlovian reward learning. Eur J Neurosci. 2010;31:1312–21. 20345912 10.1111/j.1460-9568.2010.07153.x PMC2914557

[56] Piantadosi SC, Ahmari SE. Using optogenetics to dissect the neural circuits underlying OCD and related disorders. Curr Treat Options Psychiatry. 2015;2:297–311. 31867154 10.1007/s40501-015-0056-3 PMC6924629

[57] LeBlanc KH, Maidment NT, Ostlund SB. Repeated cocaine exposure facilitates the expression of incentive motivation and induces habitual control in rats. PLoS ONE. 2013;8:e61355. 23646106 10.1371/journal.pone.0061355 PMC3640016

[58] Hermer-Vazquez L, Hermer-Vazquez R, Rybinnik I, Greebel G, Keller R, Xu S, et al. Rapid learning and flexible memory in “habit” tasks in rats trained with brain stimulation reward. Physiol Behav. 2005;84:753–9. 15885252 10.1016/j.physbeh.2005.03.007

[59] Feil J, Sheppard D, Fitzgerald PB, Yücel M, Lubman DI, Bradshaw JL. Addiction, compulsive drug seeking, and the role of frontostriatal mechanisms in regulating inhibitory control. Neurosci Biobehav Rev. 2010;35:248–75. 20223263 10.1016/j.neubiorev.2010.03.001

[60] De Kleine RA, Hutschemaekers MHM, Hendriks GJ, Kampman M, Papalini S, Van Minnen A, et al. Impaired action-safety learning and excessive relief during avoidance in patients with anxiety disorders. J Anxiety Disord. 2023;96:102698. 37004425 10.1016/j.janxdis.2023.102698

[61] Hirsch EC, Hunot S. Neuroinflammation in Parkinson’s disease: a target for neuroprotection?. Lancet Neurol. 2009;8:382–97. 19296921 10.1016/S1474-4422(09)70062-6

[62] Redgrave P, Rodriguez M, Smith Y, Rodriguez-Oroz MC, Lehericy S, Bergman H, et al. Goal-directed and habitual control in the basal ganglia: implications for Parkinson’s disease. Nat Rev Neurosci. 2010;11:760–72. 20944662 10.1038/nrn2915 PMC3124757

[63] Hoang, IB, Munier JJ, Verghese A, Greer Z, Millard SJ, DiFazio LE, et al. A novel hypothalamic-midbrain circuit for model-based learning. [Preprint] (2023). 10.1101/2023.03.02.530856.

[64] Garbusow M, Schad DJ, Sebold M, Friedel E, Bernhardt N, Koch SP, et al. Pavlovian-to-instrumental transfer effects in the nucleus accumbens relate to relapse in alcohol dependence: PIT and alcohol relapse. Addiction Biol. 2016;21:719–31. 10.1111/adb.12243 25828702

[65] Sekutowicz M, Guggenmos M, Kuitunen-Paul S, Garbusow M, Sebold M, Pelz P, et al. Neural response patterns during pavlovian-to-instrumental transfer predict alcohol relapse and young adult drinking. Biol Psychiatry. 2019;86:857–63. 31521335 10.1016/j.biopsych.2019.06.028

[66] Sommer C, Garbusow M, Jünger E, Pooseh S, Bernhardt N, Birkenstock J, et al. Strong seduction: impulsivity and the impact of contextual cues on instrumental behavior in alcohol dependence. Transl Psychiatry. 2017;7:e1183–e1183. 28763064 10.1038/tp.2017.158 PMC5611726

[67] Engeland CG, Nielsen DV, Kavaliers M, Ossenkopp K-P. Locomotor activity changes following lipopolysaccharide treatment in mice: a multivariate assessment of behavioral tolerance. Physiol Behav. 2001;72:481–91. 11282131 10.1016/s0031-9384(00)00436-4

[68] Balleine BW, Peak J, Matamales M, Bertran-Gonzalez J, Hart G. The dorsomedial striatum: an optimal cellular environment for encoding and updating goal-directed learning. Curr Opin Behav Sci. 2021;41:38–44.

[69] Bradfield LA, Becchi S, Kendig MD. Striatal acetylcholine and dopamine interactions produce situation-appropriate action selection. Curr Neuropharmacol. 2023; 10.2174/1570159X21666230912093041. 10.2174/1570159X21666230912093041 PMC11097990 37702238

[70] Daw ND, Niv Y, Dayan P. Uncertainty-based competition between prefrontal and dorsolateral striatal systems for behavioral control. Nat Neurosci. 2005;8:1704–11. 16286932 10.1038/nn1560

[71] Yin HH, Knowlton BJ, Balleine BW. Lesions of dorsolateral striatum preserve outcome expectancy but disrupt habit formation in instrumental learning. Eur J Neurosci. 2004;19:181–9. 14750976 10.1111/j.1460-9568.2004.03095.x

[72] Shan Q, Ge M, Christie MJ, Balleine BW. The acquisition of goal-directed actions generates opposing plasticity in direct and indirect pathways in dorsomedial striatum. J Neurosci. 2014;34:9196–201. 25009253 10.1523/JNEUROSCI.0313-14.2014 PMC6608360

[73] Octeau JC, Chai H, Jiang R, Bonanno SL, Martin KC, Khakh BS. An optical neuron-astrocyte proximity assay at synaptic distance scales. Neuron. 2018;98:49–66.e9. 29621490 10.1016/j.neuron.2018.03.003 PMC5916847

[74] Corkrum M, Covelo A, Lines J, Bellocchio L, Pisansky M, Loke K, et al. Dopamine-evoked synaptic regulation in the nucleus accumbens requires astrocyte activity. Neuron. 2020;105:1036–1047.e5. 31954621 10.1016/j.neuron.2019.12.026 PMC7322729

[75] Zhang W, Xiao D, Mao Q, Xia H. Role of neuroinflammation in neurodegeneration development. Sig Transduct Target Ther. 2023;8:267. 10.1038/s41392-023-01486-5 PMC10336149 37433768

[76] McNally GP, Jean-Richard-dit-Bressel P, Millan EZ, Lawrence AJ. Pathways to the persistence of drug use despite its adverse consequences. Mol Psychiatry. 2023;28:2228–37. 36997610 10.1038/s41380-023-02040-z PMC10611585

