# Supplementary material for: Dorsomedial striatal neuroinflammation causes excessive goal-directed action control by disrupting astrocyte function
Source: Neuropsychopharmacology. 2025 Sep 27;51(2):486–96. doi: 10.1038/s41386-025-02247-4 (PMC12708733; doi:10.1038/s41386-025-02247-4)
Supplement: Supplementary file 1 — Supplemental Material for manuscript [file 41386_2025_2247_MOESM1_ESM.docx]

**SUPPLEMENTARY MATERIAL**

**MATERIALS AND METHODS**

***Experiment 1 and 2: Effects of neuroinflammation in pDMS and NAc core on goal-directed action control***

*Animals and housing conditions*

For behavioural experiments, a total of 176 Long-Evans rats [34 for Experiment 1 (15 male and 19 female), 31 for Experiment 2 (15 male, 16 female), 54 for Experiment 3 (27 male and 27 female), 16 for Experiment 4 (6 male, 6 female), and 42 for Experiment 5 (20 male and 22 female)], weighing 180–350 g, 8-10 weeks of age at the beginning of the experiment were purchased from the Australian Research Centre, Perth, Australia, and were housed in groups of 2-3 in transparent amber plastic boxes located in a temperature- and humidity-controlled room with a 12-h light/dark (07:00–19:00 h light) schedule. Experiments were conducted during the light cycle. Before the experiments, all animals were habituated to the laboratory settings for a week with full access to food and water and environmental enrichment which include plastic tunnel, shreds of paper, and wooden object to gnaw. Throughout the training and actual experiment, animals were maintained at ~85% of their free-feeding body weight by restricting their food intake to 8-14g of their maintenance diet per day. All procedures were approved by the Ethics Committees of the Garvan Institute of Medical Research Sydney (AEC 18.34), and Faculty of Science, University of Technology Sydney (ETH21-6657), and the University of Newcastle (A-2020-018).

*Surgery*

Animals were anaesthetized with isoflurane (5% induction, 2–3% maintenance) and positioned in a stereotaxic frame (Kopf Instruments). An incision was made into the scalp to expose the skull surface, and the incisor bar was adjusted to align bregma and lambda on the same horizontal plane. Small holes were drilled into the skull above the appropriate targeted region and animals received bilateral injections by infusing 1 µl per hemisphere of LPS (5ug/ µl) via a 1-µl glass syringe (Hamilton Company) connected to an infusion pump (Pump 11 Elite Nanomite, Harvard Apparatus) into the pDMS (anteroposterior, −0.2mm; mediolateral, ±2.4mm (male), ±2.3mm (female); and dorsoventral, −4.5mm, relative to bregma) and another cohort of animals received LPS injected into their NAc core (anteroposterior, 1.4mm; mediolateral, ±2.2mm; and dorsoventral, −7.5mm, relative to bregma). The infusion was conducted at a rate of 0.15 µl/min, and injectors were left in place for an additional 5 min to ensure adequate diffusion and to minimize LPS spread along the injector tract. The remaining control animals underwent identical procedures but with injection of sterile saline rather than LPS. A nonsteroidal anti-inflammatory/antibiotic agent were administered preoperatively and postoperatively to minimize pain and discomfort. Animals were allowed to recover for 7 days before the onset of any behavioural training.

*Apparatus*

All behavioural procedures took place in twelve identical sound attenuating operant chambers (Med Associates, Inc.,) and these chambers were located within individual cubicles. The ceiling, back wall, and hinged front door of the operant chambers were made of a clear Plexiglas and the side wall were made of grey aluminium. The floor was made of stainless steel grids. Each chamber was equipped with a recessed food magazine, located at the base of one end wall, through which 20% sucrose-10% polycose solution (0.2 ml) and food pellets (45 mg; Bio-Serve, Frenchtown, NJ) could be delivered using a syringe pump and pellet dispenser, into separate compartments respectively. Two retractable levers could be inserted individually on the left and right sides of the magazine. An infrared light situated at the magazine opening was used to detect head entries. Illumination was provided by a 3-W, 24-V houselight situated at the top-centred on the left end wall opposite the magazine provided constant illumination, and an electric fan fixed in the shell enclosure provided background noise (≈70 dB) throughout training and testing. The apparatus was controlled, and the data were recorded using Med-PC IV computer software (Med Associates, Inc.). The boxes also contained a white-noise generator, a sonalert that delivered a 3 kHz tone, and a solanoid that, when activated, delivered a 5 Hz clicker stimulus. All stimuli were adjusted to 80 dB in the presence of background noise of 60 dB provided by a ventilation fan. Outcome devaluation procedures took place in transparent plastic tubs that were smaller, but otherwise identical to the cages in which rats were housed.

*Food restriction and Chow maintenance*

One week following recovery from surgery, animals underwent 3 days of food restriction before the onset of lever press training. During this time animals received 10-14g of chow per day, and their weight was monitored daily to ensure it remained at ~85% of their pre-surgery body weight. For the initial Pavlovian and instrumental training, as well as the first round of testing for sPIT, devaluation, and reinstatement, the chow that rats were maintained on the higher-fat, higher-protein Gordons Specialty Feed (see Table 1). Following reinstatement testing, animals were switched to a lower fat, lower protein Irradiated Specialty Feed’s chow (see Table 1) and re-trained and re-tested.

*Pavlovian training*

For the first 8 days, animals were placed in operant chambers for 60 min during which they received eight 2 min presentations of two conditioned stimuli (CS; white noise or clicker) paired with one of two outcomes (sucrose solution or pellet) presented on a random time schedule around an average of 30 s throughout each CS presentation. Each CS was presented 4 times, with a variable intertrial interval (ITI) that averaged to 5 min. For half the subjects, tone was paired with sucrose and noise with pellets, with the other half receiving the opposite arrangement. Magazine entries throughout the session were recorded and reported for the 2 min prior to each CS presentation (PreCS) and the 2 min during each CS presentation.

*Lever press training*

Following Pavlovian training, animals were trained to press a left and right lever over 8 days which earned the same sucrose and grain pellet outcomes. Specifically, for half of the animals, the left lever earned pellets and the right lever earned sucrose, and the other half received the opposite arrangement (counterbalanced). Each session lasted for 50 minutes and consisted of two 10 minutes sessions on each lever (i.e., four x 10 minutes sessions in total) separated by a 2.5 minutes time-out period in which the levers were retracted and the houselight was switched off. Animals could earn a maximum of 40 sucrose and 40 pellets deliveries within the session. For the first 2 days, animals were trained on a continuous reinforcement schedule (CRF) in which each lever press produced a single outcome. Animals were then shifted to a random ratio-5 schedule for the next 3 days (i.e. each action delivered an outcome with a probability of 0.2), then to a RR-10 schedule (or a probability of 0.1) for the final 3 days. After 40 sucrose solutions and 40 pellets were delivered or 50 minutes had elapsed, whichever came first, the session was terminated, levers were retracted, and house lights switched off.

*Pavlovian Instrumental Transfer (Specific PIT) test*

One day after the end of instrumental training, rats were tested for sPIT performance. For this test, responding on both levers was first extinguished for 8 min to reduce baseline performance. Subsequently, each CS was presented four times over the next 40 min in the following order: clicker-noise-noise-clicker-noise-clicker-clicker-noise. Each CS lasted 2 min and had a fixed ITI of 3 min. Magazine entries and lever pressing rates were recorded throughout the session and responses were separated into PreCS and CS periods (2 min each). Lever presses were recorded but not reinforced.

*Outcome Devaluation*

One day after sPIT testing, rats were given 1 day of instrumental retraining on RR-10 in the manner previously described. On the following day, animals were given free access to either the pellets (20 g placed in a bowl) or the sucrose solution (100 ml in a drinking bottle) for 1 hr. The amount of pellets and sucrose solution consumed each day was measured. Animals were then placed in the operant chamber for a 10 min choice extinction test. During this test, both levers were extended and lever presses recorded, but no outcomes were delivered. The next day, a second devaluation test was administered with the opposite outcome (i.e. if animals were prefed on pellets the previous day they were now prefed on sucrose, and vice versa). Following pre-feeding animals were again placed into the operant chambers for a second 10 min choice extinction test. All test results are reported as averaged across these two tests.

*Outcome Selective Reinstatement Test*

After devaluation testing, rats received one day of instrumental retraining on an RR-10 schedule for 1 day. The next day, animals were tested for outcome-selective reinstatement in which rats received a 15 min period of extinction to reduce baseline performance. They then received four reinstatement trials separated by 4 min each as before, and each reinstatement trial consisted of a single free delivery of either the sucrose solution or the grain pellet presented in the following order: sucrose, pellet, pellet, and sucrose. Responding was measured during the 2 min periods immediately before (pre) and after (post) each delivery.

*Switching maintenance chow, re-training and re-testing*

As noted, a highly palatable home chow (the high-fat/high-protein Gordan’s chow, specifications shown in Table 1) was initially used for Experiment 1 to reduce performance in Sham controls. Following training and testing during which animals were given this chow, rats were switched to a smaller amount (6-8g) of less palatable home chow (lower-fat/lower-protein Specialty Feed’s chow, specifications shown in Table 1) to increase hunger and motivation to lever press for food, with the aim of improving test performance in group Sham.

Following the switch from Gordon’s to Specialty feeds chow, rats were given an additional 4 days of Pavlovian training, and an additional 4 days of intrumental training, then tested for performance on sPIT, outcome devaluation, and outcome-selective reinstatement as before.

*Tissue preparation*

One day after the outcome-selective reinstatement test, animals were sacrificed via CO2 inhalation and perfused transcardially with cold 4% paraformaldehyde in 0.1 M phosphate buffer saline (PBS; pH 7.3-7.5). Brains were rapidly and carefully removed and postfixed in 4% paraformaldehyde overnight and then placed in 30% sucrose. Brains were sectioned coronally at 40 µm through the pDMS and NAc core defined by Paxinos and Watson (2014) using a cryostat (CM3050S, Leica Microsystems) maintained at approximately -20˚Celsius. The sectioned slices were immediately immersed in cryoprotectant solution and stored in the -20°C freezer.

Later, five representative sections from pDMS and NAc core were selected for each rat. Sections were first washed three times (10 minutes per wash) in PBS to remove any exogenous substances. The sections were then incubated in a blocking solution comprising of 3% Bovine Serum Albumin (BSA) + 0.25% TritonX-100 in 1 x PBS for one hour to permeabilize tissue and block any non-specific binding. Sections were then incubated in anti-GFAP mouse primary antibody (1:300, Cell Signalling Technology Catalog #3670), anti-IBA1 rabbit primary antibody (1:500, FUJIFILM Wako Chemicals U.S.A. Corporation), and anti-NeuN chicken primary antibody (1:1000, GeneTex Catalog #GTX00837) diluted in blocking solution for 72 h at 4°C. Sections were then washed 3 times in 1 × PBS and incubated overnight at 4°C in goat anti-mouse AlexaFluor-488 secondary antibody (1:250, ThermoFisher Catalog #A-11001), donkey anti-rabbit AlexaFluor-568 secondary antibody (1:250, ThermoFisher Catalog #A10042), and goat anti-chicken AlexaFluor-647 secondary antibody (1:250, ThermoFisher Catalog #A-21449), followed by a counterstain with 4′,6-diamidino-2-phenylindole (DAPI; Thermo Scientific; 1:1000, diluted in 1x PBS). Finally, every section was mounted onto Superfrost microscope slides (Fisher Scientific) and were coverslipped (Menzel-Glaser) using the mounting agent Vectashield and left to dry overnight in darkness.

*Imaging and immunofluorescence analysis*

For quantification of GFAP, IBA1, and NeuN, a single image was taken of the pDMS and NAc core per hemisphere of each slice (6-10 images in total per brain region of each rat) on a Nikon TiE2 microscope using a 10x objective and Leica STELLARIS 20x air objective for representative images.

*Microscopy:* Images were quantified using imaging software (ImageJ, Fiji Cell Counter), whereby each fluorescent channel was split to isolate and count the cells of interest. Z-stacks were used instead of simply a single image plane. Briefly, the image was adjusted to 8-bit and background subtraction was applied to remove background noise. Thresholding was used to isolate positive stained cells and the threshold for contrast and brightness was adjusted for all images until consistent between images (maximum: 255, minimum: 0). Images were then converted to binary and finally, the Analyze Particles tool was used to quantify the number of cells based on a minimum particle size of 16. ImageJ counted each cell between our parameters and presented it as a “count.” Circularity and perimeter measurement are both part of the Analyze particles plug-in in ImageJ. This was followed by intensity measurement, which is represented as Mean grey value (MGV) and background intensity subtracted from the reported MGV.

*Data and Statistical analysis*

Data were collected automatically by Med-PC and uploaded to Microsoft Excel using Med-PC to Excel software. Pavlovian conditioning and lever press acquisition data was analysed using two-way repeated measures ANOVAs controlling the per-family error rate at α=0.05. If conditions for sphericity were not met, the Greenhouse-Geisser correction was applied. To allow for a more fine-grained analysis of test data, all data for sPIT, outcome devaluation, and outcome-selective reinstatement were analysed using complex orthogonal contrasts controlling the per-contrast error rate at α=0.05 according to the procedure described by Hays (*21*). Acquisition data were expressed as mean ± standard error of the mean (SEM) averaged across counterbalanced conditions. Test data were expressed as individual data points with means. If interactions were detected, follow-up simple effects analyses (α=0.05) were calculated to determine the source of the interaction. For immunohistochemical analysis, counts and intensity were compared between LPS and Sham groups using two tailed t-tests and correlated using GraphPad. Test behaviours were correlated with GFAP, IBA1, and NeuN using the immunohistochemical results from Figure 3. For correlations with behaviour we used a “PIT score”, a “devaluation score”, or a “reinstatement score” that were calculated in such a way as to ensure that any association detected was not driven by baseline differences in lever press responding *per se*, but rather by the animals selectivity of responding for one or the other levers. For these scores, we first calculated suppression ratio (SR) scores on each of the levers individually (i.e., the same and different levers for sPIT, the valued and devalued levers for devaluation, and the reinstated and non-reinstated levers for outcome selective reinstatement) according to Equation 1:

1. $Score=\frac{Lever Press rate on Test}{Lever Press rate on Test + Baseline Lever Press Rate}$

In this equation, “baseline lever press rate” was taken as the average press rate on each lever across the last two days of training prior to test. We then calculated the PIT score by subtracting the normalised scores on the different lever from the normalised scores on the same lever (i.e. Same – Different), such that a higher score indicated better sPIT performance. Likewise, for devaluation we subtracted the normalised scores on the devalued from those on the valued lever (i.e. Valued – Devalued), such that a higher score indicated better devaluation performance, and did the same thing for reinstatement, this time subtracting scores on the nonreinstated from scores on the reinstated lever (i.e. Reinstated – NonReinstated) such that a high score indicated better reinstatement performance. Each of these scores were then separately correlated with GFAP, IBA1, and NeuN counts (correlations with GFAP, IBA1, and NeuN intensity can be found in th excel files uploaded online DOI 10.17605/OSF.IO/297ZU). Values of p < 0.05 were considered statistically significant. The statistical software GraphPad Prism, SPSS, and PSY were used to carry out these analyses.

***Experiment 3: Effects pDMS neuroinflammation on overtraining-induced habits***

*Surgery*

All surgical procedures were conducted identically to that described for Experiment 1.

*Food restriction and Chow maintenance*

For this experiment, animals received only 6-8g of the Irradiated Specialty Feeds chow per day to maintain high motivation conditions. They did not receive Gordon’s chow at any point.

*Apparatus*

All Apparatus were as described for Experiments 1 and 2.

*Magazine Training*

Following recovery from surgery to inject LPS or saline into the pDMS, animals received 3 days of food deprivation and were then given two sessions of magazine training. For these sessions, the house light was turned on at the start of the session and turned off when the session was terminated. No levers were extended. Sucrose solution was delivered at random 60 s intervals for 30 outcomes per session. The session terminated after 45 min or after 30 outcomes had been delivered, whichever came first.

*Lever Press Training*

Following magazine training, animals then received 8 days of instrumental training (two sessions per day) to press a single lever for sucrose solution delivery. Animals received three sessions of continuous reinforcement, four sessions of random interval of 15 s (RI-15), four sessions of RI-30, and four sessions of RI-60. Right and left lever assignment was counterbalanced across animals. Sessions ended, levers retracted and the houselight terminated when 30 reinforcements were earned or after 60 min, which ever came first.

*Progressive ratio test*

Following lever press training, animals underwent 2-h of progressive ratio (PR) testing each day for 3 days. A progressive ratio schedule requires the subject to perform an increasing number of lever presses for the next presentation of a reinforcer (Hodos, 1961). For the current study, the PR was set at n+5. This meant that animals initially received a sucrose reward for a single lever press, then for 5 lever presses, then n+5 lever presses until breakpoint – with breakpoint defined as 5 min of no lever pressing. The number of responses required to obtain each successive delivery of the sucrose reward was collected automatically by Med-PC.

*Outcome devaluation*

The day after progressive ratio testing, animals were given 2 days of instrumental retraining on an RI-60 schedule in the manner previously described. The following day, the sucrose solution was devalued using conditioned taste aversion method for half of the animals. That is, all animals were given ad libitum access to sucrose solution in clear plastic tubs for 30 min each day for 3 days. Immediately after the 30 mins, half of each type of lesion group received an intraperitoneal injection of lithium chloride (0.15 M LiCl, 20 ml/kg) to induce illness which the rat will associate with the outcome, effectively devaluing it, after which they placed back in their home cages. The remaining rats received 0.9% purified saline injections (20 ml/kg) and these animals comprised the valued groups. In total this manipulation yielded 4 groups: Sham-Valued, Sham-Devalued, LPS-Valued, LPS-Devalued. The amount of sucrose solution consumed each day was measured.

*Extinction test*

The day following the last day of LiCl pairings, all animals received a 5 min extinction test. The test began with the insertion of the same lever used during training and ended with the retraction of the lever. Lever presses were recorded, and no sucrose reward was delivered.

*Tissue Processing and Fluorescent Microscopy*

All tissue processing and microscopy were conducted identically to that described for Experiments 1 and 2.

*Statistical analysis*

Lever press and magazine entry data were collected automatically by Med-PC (version 5) and uploaded directly to Microsoft Excel using Med-PC to Excel software. Lever press acquisition and progressive ratio data were analysed using repeated measures (Group x Session) ANOVA controlling the per-family error rate at α=0.05. To allow for a more fine-grained analysis of test data, we used planned, complex orthogonal contrasts controlling the per-contrast error rate at α=0.05 for analyzing the outcome devaluation according to the procedure described by Hays (1973). The amount of sucrose consumed was analysed using Three-Way ANOVA repeated measures. If conditions for sphericity were not met, the Greenhouse-Geisser correction was used. Data analysis was conducted in the manner described for Experiment 3.

***Experiment 4: Patch-clamp electrophysiology***

*Acute brain slice preparation*

To prepare brain slices animals were deeply anesthetised using ketamine injection (100mg/kg, i.p.) and then rapidly decapitated. Following this, brains were rapidly extracted and immersed in ice-cold sucrose substituted artificial cerebrospinal fluid (ACSF) containing (in mM): 236 sucrose, 25 NaHCO_3_, 11 glucose, 2.5 KCL, 1 NaH2PO4, 1 MgCl2, and 2.5 CaCl2. Coronal slices (300µm) of the pDMS were made using a vibrating microtome (VT1200s, Leica, Nussloch, Germany). Slices were then transferred to an incubation chamber containing oxygenated ACSF (120mM NaCL substituted for sucrose) and allowed to equilibrate for 1-hour at room temperature (22-24°C) prior to recording.

*Patch-clamp electrophysiology*

Slices were transferred to a recording chamber and continuously perfused at a rate of 4-6 bath volumes/min with ASCF constantly bubbled with Carbonox (95% O_2_, 5% CO_2_) to achieve a final pH of 7.3-7.4. All recordings were obtained at room temperature (22-24°C), with neurons visualized using near-infrared differential interference contrast optics (IR-DIC). Recordings were restricted to the pDMS in both LPS and hM4Di-DREADD studies, and taken using patch pipettes (4-8 MW, Harvard Glass) filled with a potassium gluconate based internal solution containing (in mM): 135 C_6_H_11_KO_7_, 8 NaCL, 2 Mg_2_-ATP, 10 HEPES, 0.1 EGTA, and 0.3 Na_3_GTP, pH 7.3 (with KOH). Recordings were collected using a Multiclamp 700B amplifier (Molecular Devices, Sunnyvale CA). Signals were sampled at 20kHz, filtered at 10kHz and digitised using an InstraTECH ITC-18 A/D board (HEKA Instruments, Belmore, New York), acquired using Axograph X software (Axograph X, Sydney, Australia). Putative MSN cell selection was based on MSN cell morphology and post-hoc confirmation of MSN delayed firing AP profile, excluding cells without this profile from analysis.

Once whole-cell recording was initiated, series and input resistance were calculated based on the response to a -5 mV voltage step from a holding potential of -70 mV. These measurements were repeated throughout and at the end of all recordings, and data were rejected if this changed by >20 % for an individual cell. Action potential discharge was investigated in current clamp mode, firstly at RMP, and subsequently voltage clamped at -80mV by injection of current when necessary. Depolarising current steps used to evoke AP discharge were increased in 20pA increments for a duration of 1 second and features relating to AP profile were extracted from this data. For recordings using hM4Di-DREADD agonist DCZ (1mM) the above recordings were firstly taken in ACSF and then repeated following bath application of DCZ. Data were analysed offline using Axograph X and Igor Pro 9 (Wavemetrics, Portland, OR) software. AP threshold was taken from rheobase response and AP characteristics were extracted from this including latency to rheobase AP rise time, AP amplitude, AP half-width, AHP peak and AHP position. AP discharge properties were then calculated from rheobase +20pA to determine frequency (mean and instantaneous) and interspike interval.

*Statistics*

Data is presented as mean ± SEM. Unpaired t-tests with Welch’s correction were used to compare LPS and sham affected MSN populations. Paired t-tests were used when comparing unaffected and DCZ treated MSNs.

***Experiment 5: Chemogenetic activation of Gi-protein-coupled receptors in astrocytes***

*Chemogenetics*

The DREADD agonist deschloroclozapine (DCZ) dihydrochloride (NIMH D-925) was acquired from National Institute of Mental Health (NIMH) through the NIMH Chemical Synthesis and Drug Supply Program. DCZ was diluted with normal saline (SAL) (0.9% w/v NaCL) to a final injectable concentration of 0.1 mg/kg (at a volume of 1ml/kg). DCZ was always handled in dim/low light conditions (i.e. a single lamp in a darkened room) and freshly prepared on the morning of each test day.

*Surgery*

All surgical procedures were conducted identically to that described for Experiment 1, except that animals received bilateral injections of 1 µl per hemisphere of AAV-GFAP-hM4Di-mCherry (*Addgene*, item ID 50479-AAV5, titer 7×10¹² vg/mL). The infusion was conducted at a rate of 0.2 µl/min, and injectors were left in place for an additional 5 min to ensure adequate diffusion and to minimize DREADDs spread along the injector tract. The remaining control animals underwent identical procedures but with injection of AAV-GFAP104-mCherry (*Addgene*, item ID 58909-AAV5, titer 1×10¹³ vg/mL) as control group.

*Apparatus and Behavioural Procedures*

All apparatus and behavioural procedures were conducted identically to that described for Experiment 1, except for outcome devaluation (specific satiety) where animals were given free access to either the pellets or the sucrose solution for 45 mins instead of 1 hr, after which DCZ was administered intraperitoneally (i.p.) and rats returned to their home cage for 25-30 min prior to behavioural testing.

*Tissue Processing and Fluorescent Microscopy*

The extent of the expression was determined using the boundaries defined by Paxinos and Watson (2014). Sections were then stained with Living Colors® DsRed Polyclonal Antibody (1:500, Takara Bio USA, Inc. Catalog #632496) to recognize the mCherry DREADDs expression, anti-GFAP mouse primary antibody (1:300, Cell Signalling Technology Catalog #3670) to check the co-localization, diluted in blocking solution for 72 h at 4°C. Sections were then washed 3 times in 1 × PBS and incubated overnight at 4°C in donkey anti-rabbit AlexaFluor-568 secondary antibody (1:500, ThermoFisher Catalog #A10042), goat anti-mouse AlexaFluor-488 secondary antibody (1:500, ThermoFisher Catalog #A11001), followed by a counterstain with DAPI (Thermo Scientific; 1:1000, diluted in 1x PBS). Sections were mounted and quantified using procedures identical to those described above.

*Statistical analysis*

All statistical analysis was conducted identically as described for Experiment 1.

**Tables**

|  | Gordons Specialty Feed | Irradiated Specialty Feed |
| --- | --- | --- |
| Protein | 23% | 19% |
| Saturated Fat | 21.3% | 0.78% |
| Mono-unsaturated Fat | 42.9% | 2.06% |
| Poly-unsaturated Fat | 30.7% | 1.88% |
| Crude Fibre | 5% | 5.20% |

***Table 1. Nutritional information of the lab chow used during the experiment***

| **Measurement** | **LPS** | **Saline** | **Significant Difference** |
| --- | --- | --- | --- |
| Amplitude (mV) | 67.37 | 71.09 | ns (0.1989) |
| **Rise (ms)** | **0.8204** | **0.7286** | *** (0.0489)** |
| Width (ms) | 2.424 | 2.309 | ns (0.3305) |
| AHP Peak (mV) | -17.33 | -15.48 | ns (0.0577) |
| AHP Position (ms) | 16.85 | 17.33 | ns (0.8014) |
| AHP Threshold (mV) | -35.79 | -37.82 | ns (0.1562) |
| Rheobase (pA) | 196.7 | 222.0 | ns (0.4996) |
| Latency Rheobase (ms) | 608.3 | 621.5 | ns (0.8741) |
| Resting Membrane Potential (mV) | -73.30 | -72.81 | ns (0.8514) |
| **Instantaneous**  **Frequency (Hz)** | **6.052** | **4.760** | *** (0.0359)** |
| Average Frequency (Hz) | 3.091 | 3.200 | ns (0.8142) |
| **Interspike Interval (ms)** | **177.8** | **265.0** | *** (0.0300)** |

**Table 2: Full results for firing properties of medium spiny neurons in posterior dorsomedial striatum injected with 1ul lipopolysaccharide (LPS, 5mg/mL) at resting membrane potential.**

| **Measurement** | **LPS** | **Saline** | **Significant Difference** |
| --- | --- | --- | --- |
| **Amplitude (mV)** | **64.54** | **72.80** | *** (0.0105)** |
| **Rise (ms)** | **0.8743** | **0.7086** | **** (0.0027)** |
| Width (ms) | 2.552 | 2.389 | ns (0.2822) |
| **AHP Peak (mV)** | **-17.24** | **-14.26** | **** (0.0020)** |
| AHP Position (ms) | 17.61 | 17.95 | ns (0.8497) |
| **AHP Threshold (mV)** | **-36.16** | **-40.67** | *** (0.0228)** |
| Rheobase (pA) | 256.3 | 272.7 | ns (0.6721) |
| Latency Rheobase (ms) | 524.1 | 661.4 | ns (0.0778) |
| Instantaneous  Frequency (Hz) | 6.340 | 5.422 | ns (0.3581) |
| Average Frequency (Hz) | 2.545 | 3.067 | ns (0.3126) |
| Interspike Interval (ms) | 238.6 | 230.1 | ns (0.8289) |

**Table 3: Full results for firing properties of medium spiny neurons in posterior dorsomedial striatum injected with 1ul lipopolysaccharide (LPS, 5mg/mL) at -80mV.**

| **Measurement** | **ACSF** | **DREADDs** | **Significant Difference** |
| --- | --- | --- | --- |
| Amplitude (mV) | 75.39 | 75.30 | ns (0.9809) |
| Rise (ms) | 0.7501 | 0.7640 | ns (0.8687) |
| Width (ms) | 2.764 | 3.074 | ns (0.3844) |
| AHP Peak (mV) | -14.95 | -12.07 | ns (0.0879) |
| AHP Position (ms) | 23.59 | 22.64 | ns (0.5459) |
| **AP Threshold (mV)** | **-40.39** | **-45.04** | *** 0.0118** |
| **Rheobase (pA)** | **185.7** | **125.7** | **** 0.0028** |
| Latency Rheobase (ms) | 640.5 | 441.5 | ns (0.2462) |
| **Resting Membrane Potential (mV)** | **-76.19** | **-64.67** | **** (0.0018)** |
| Instantaneous  Frequency (Hz) | 4.762 | 4.749 | ns (0.9910) |
| Average Frequency (Hz) | 3.429 | 2.429 | ns (0.1563) |
| Interspike Interval (ms) | 227.5 | 259.5 | ns (0.5656) |

**Table 4: Full results for firing properties of medium spiny neurons adjacent to hM4Di-expressing astrocytes in posterior dorsomedial striatum at resting membrane potential.**

| **Measurement** | **ACSF** | **DREADDs** | **Significant Difference** |
| --- | --- | --- | --- |
| Amplitude (mV) | 79.78 | 79.49 | ns (0.9293) |
| Rise (ms) | 0.6602 | 0.6550 | ns (0.8425) |
| Width (ms) | 2.552 | 2.937 | ns (0.1000) |
| AHP Peak (mV) | -13.77 | -11.19 | ns (0.4693) |
| AHP Position (ms) | 21.98 | 19.39 | ns (0.1974) |
| AP Threshold (mV) | -43.85 | -48.04 | ns (0.3269) |
| Rheobase (pA) | 203.3 | 220.0 | ns (0.4859) |
| Latency Rheobase (ms) | 611.2 | 604.2 | ns (0.9769) |
| Instantaneous  Frequency (Hz) | 4.478 | 3.664 | ns (0.3537) |
| Average Frequency (Hz) | 3.400 | 2.200 | ns (0.0705) |
| Interspike Interval (ms) | 245.2 | 367.8 | ns (0.4086) |

**Table 5: Full results for firing properties of medium spiny neurons adjacent to hM4Di-expressing astrocytes in posterior dorsomedial striatum at -80mV.**

**Supplemental Figures and Results**

**
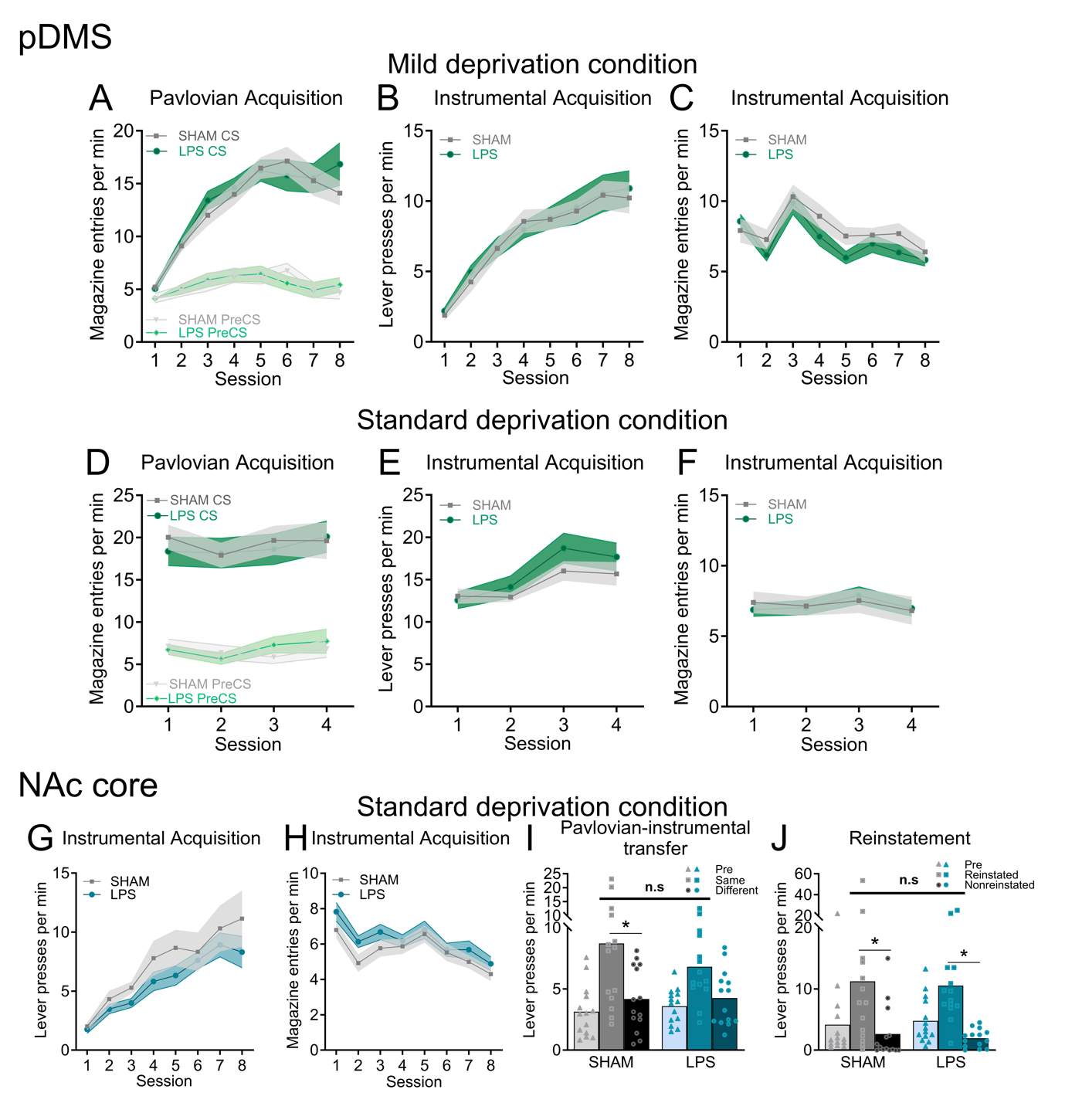
**

**Supplemental Figure 1, relates to Figure 1. Supplemental behaviour results.** *There were no significant Sham/LPS differences at any stage of acquisition for pDMS experiment (Experiment 1). (A-C) Acquisition under mild deprivation conditions, (A)* *Magazine entries per min* *(±SEM) during Pavlovian conditioning, F (7,196) = 0.669, p = 0.698, for CS x group x session interaction, (B) Lever presses per min (±SEM) during instrumental conditioning, main effect of day F (7,196) = 53.28, p < 0.001, no main effect of group and no day x group interaction,* *all Fs < 1,(C) Magazine entries per min (±SEM) during instrumental conditioning, main effect of day F (7,196) = 15.282, p < 0.001, no main effect of group and no day x group interaction, all Fs < 1, (D-E) Acquisition under standard deprivation conditions, (D) Magazine entries per min (±SEM) during Pavlovian conditioning, F (3,84) = 2.15, p = 0.111, for CS x group x session interaction, (E)* *Lever presses per min (±SEM) during instrumental conditioning, main effect of day F (3,84) = 15.87, p < 0.001, no main effect of group and no day x group interaction, all Fs < 1, (F) Magazine entries per min (±SEM) during instrumental conditioning, main effect of day F (3,84) = 2.865, p = 0.041, no main effect of group and no day x group interaction, all Fs < 1. (G-J) Supplemental behavioural results from NAc core neuroinflammation study, there were no significant Sham/LPS differences in instrumental responding for this experiment, (G) Lever presses per min (±SEM) during instrumental conditioning, main effect of day F (7,182) = 27.711, p < 0.001, no main effect of group and no day x group interaction, Fs < 1, (H) Magazine entries per min (±SEM) during instrumental conditioning, main effect of day F (7,182) = 9.692, p < 0.001, no main effect of group and no day x group interaction, Fs < 1, (I) Individual data points and mean magazine entries per min during Pavlovian instrumental transfer testing, a main effect of sPIT, F (1,26) = 14.349, p = .001, that did not interact with the group, F (1,26) = 1.118, p = 0.30. A significant simple effect for the Sham group (Same > Different), F (1,26) = 11.739, p = 0.002, but no such effect (or a marginal simple effect) for the LPS group (Same = Different), F (1,26) = 3.728, p = 0 .064, (J) Individual data points and mean magazine entries per min during outcome selective reinstatement testing, a main effect of reinstatement (Reinstated > Nonreinstated) F (1,26) = 19.278, p < 0.001, which did not interact with any group differences, all Fs < 1.* denotes p < 0.05.*

*
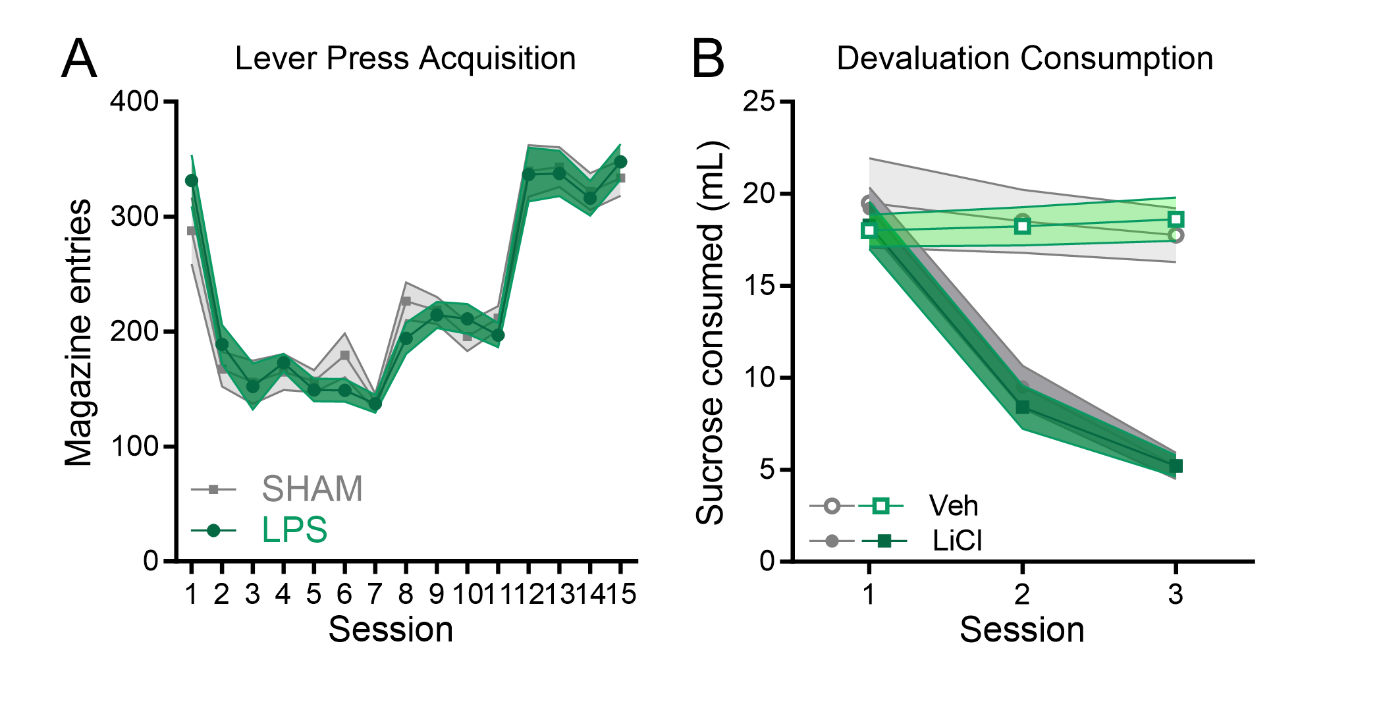
*

**Supplemental Figure 2, relates to Figure 2. pDMS neuroinflammation did not alter magazine entries during lever press acquisition, nor sucrose devaluation by conditioned taste aversion.** *(A) Magazine entries per min* *(±SEM) during instrumental conditioning, main effect of day F (14,546) = 51.27, p < 0.001, no Sham/LPS difference, F (1,39) = 0.002, p = 0.9629, and no day x group interaction, F < 1, (B) Millilitres of sucrose consumed (±SEM) during conditioned taste aversion training, session x devaluation interaction, F (1.64,60.9) = 90.44, p < 0.001 that did not interact with group, F < 1.*

*
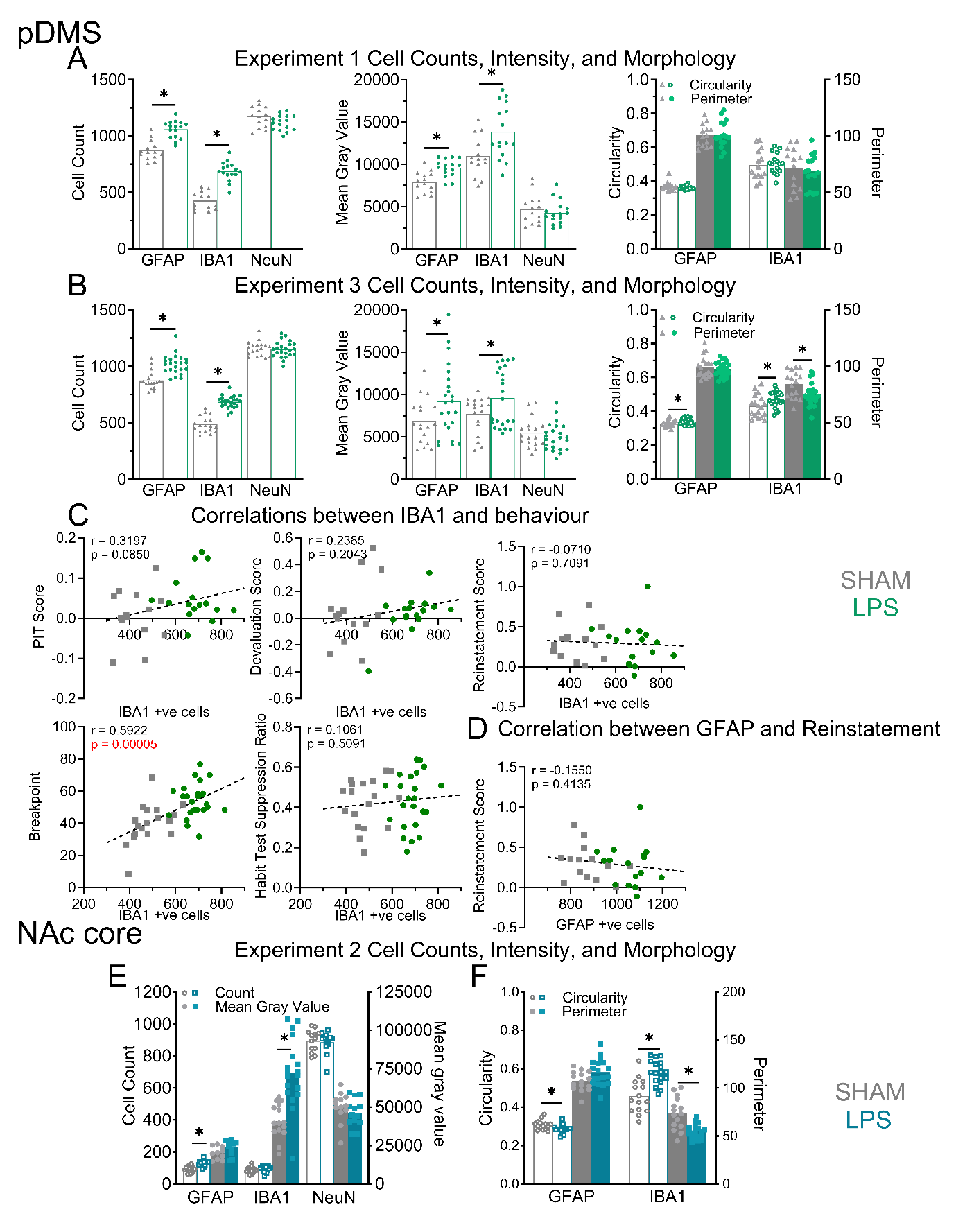
*

**Supplemental Figure 3. Relates to Figure 3. Supplemental immunohistochemical results following** i**njections of lipopolysaccharide (LPS) into posterior dorsomedial striatal (pDMS) in Experiments 1&3.** *(A-B) Individual data points and mean values for quantification of, from left to right, cell counts, mean gray value, circularity (left y axis), and perimeter (right y axis) of GFAP, IBA1, and NeuN from rats in (A) Experiment 1 and (B) Experiment 3. For Experiment 1: cell counts were significantly higher in LPS tissue relative to Sham for GFAP, t(28) = 6.255, p < 0.001, and IBA1, t(28) = 8.74, p < 0.001, but not for NeuN, t(28) = 1.9, p = .068, mean gray value was likewise significantly higher in LPS tissue relative to Shams for GFAP, t(28) = 4.046, p < 0.0014, IBA1, t(28) = 2.799, p = 0.0092, but not for NeuN, t(28) = 0.7616, p = 0.4527, circularity and perimeter of cells did not differ for any GFAP-positive or IBA1-positive cells in Experiment 1, closest t(28) = 0.77, p = 0.443, for IBA1 perimeter. For Experiment 3: cell counts were significantly higher in LPS tissue relative to Sham for GFAP, t(39) = 5.38, p < 0.001, and IBA1, t(39) = 10.27, p < 0.001, but not for NeuN, t(39) = 0.336, p = 0.074, mean gray value was likewise significantly higher in LPS tissue relative to Shams for GFAP, t(39) = 2.072, p = 0.045, IBA1, t(39) = 2.088, p = 0.043, but not for NeuN, t(39) = 0.8789, p = 0.3848, GFAP-positive cells were significantly more circular for this experiment, t(39) = 2.152, p = 0.0378, as were IBA1-positive cells, t(39) = 2.109, p = 0.0414, whereas perimeter of GFAP-positive cells did not differ between groups, t(39) = 0.7562, p = 0.4541, but was significantly lower in IBA1-positive cells for LPS animals, t(39) = 2.665, p = 0.0113. (C-D) Correlations between (C) IBA1 and (D) GFAP and behavioural performances, r and p values displayed on graphs. (E-F) Results of analyses of immunohistochemical labelling of GFAP, IBA1, and NeuN following NAc core neuroinflammation, (E) Individual data points for cell counts (open shapes and left y axis) and Mean Gray Value (closed shapes and right y axis) values for GFAP (increased counts, t(26) = 4.886, p < 0.001, but not intensity, t(26) = 0.3273, IBA1 (increased intensity, t(26) = 5.110, p < 0.001, but not counts, t(26) = 0.9705, p = 0.5238, and NeuN-positive cells (did not differ between groups), (F) Individual data points for circularity (open shapes and left y axis) and perimeter (closed shapes and right y axis) for GFAP (significant decrease in GFAP circularity, t(26) = 2.412, p = 0.047; perimeter was unchanged, t(26) = 1.955, p = 0.1209) and IBA1 (significant increase in IBA1 circularity, t(26) = 3.829, p = 0.0024; significantly smaller perimeter, t(26) = 3.502, p = 0.0055). * denotes that the p < 0.05.*


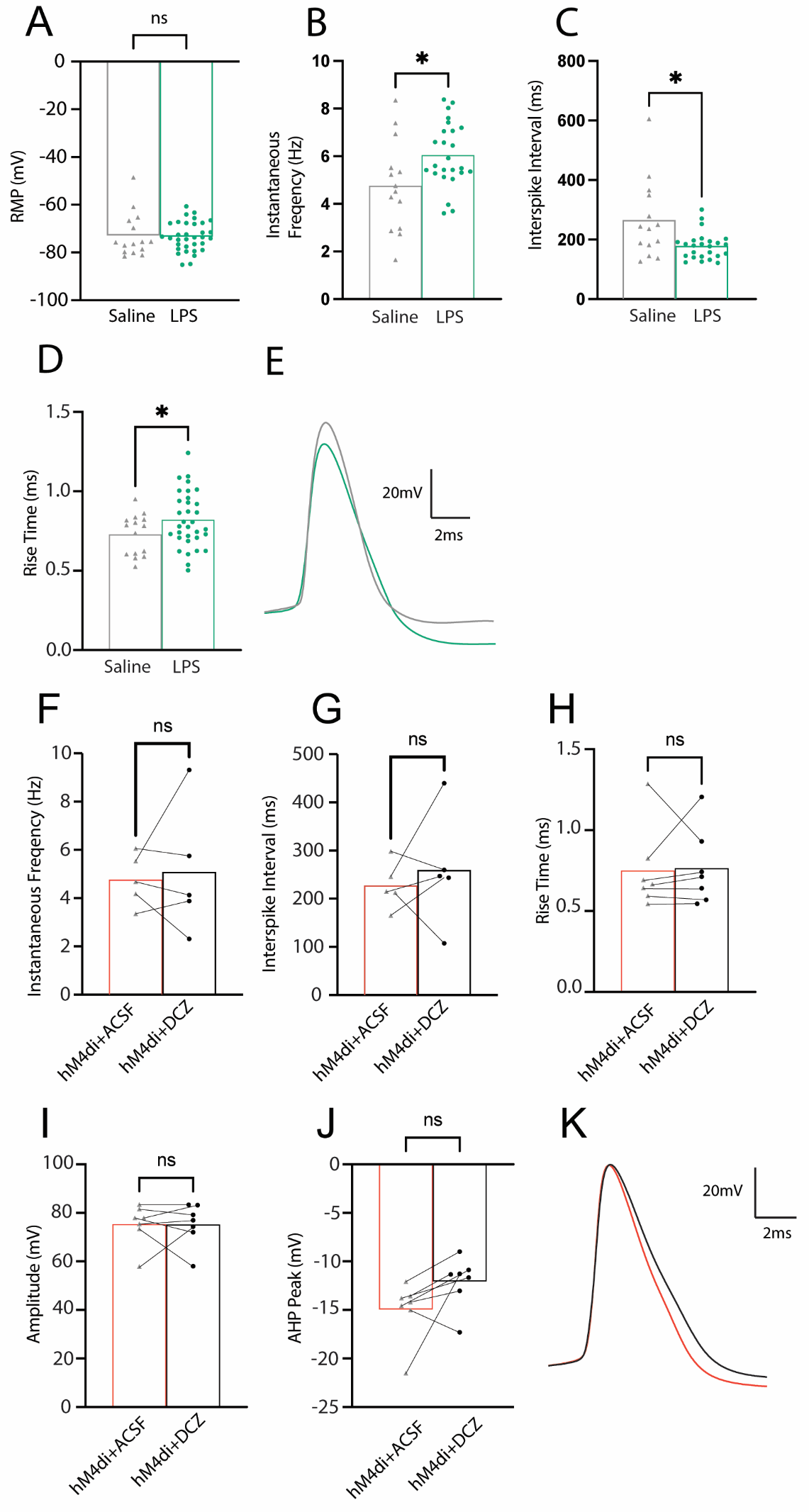


**Supplement Figure 4. Relates to Figure 4.** *(A-L) Supplemental data from whole-cell patch clamp electrophysiology recordings of MSNs following (A-E) LPS or sham injections into the pDMS or (F-K) following the application of hM4Di-DREADD agonist DCZ to transfected astrocytes. (A-E) Individual data points and means from recordings taken at resting membrane potential (RMP) showing (A) no change to RMP, (B) increased rise time (t36.26 = 2.038, p = 0.0489), (C) increased instantaneous frequency (t20.47 = 2.245, p = 0.0359), and (D) reduced interspike interval (t16.23 = 2.417, p = 0.0278). Rise time changes are further reflected in (E) example cell average traces for the AP profile characteristics (LPS = green, saline = grey). Individual data points and means following DCZ application with MSNs voltage clamped at -80mV (F-K). Data reflected in example cell profile traces presented in (H; ASCF = red, DCZ = black) show AP profile characteristics of rise time and amplitude. LPS vs saline; LPS at RMP n = 33 cells and at -80 voltage clamp n = 32 cells, from n = 4 animals; saline; n = 15 cells from n = 3 animals. GFAP-HM4Di n = 7 cells from n = 2 animals tested with ACSF then DCZ.*

*
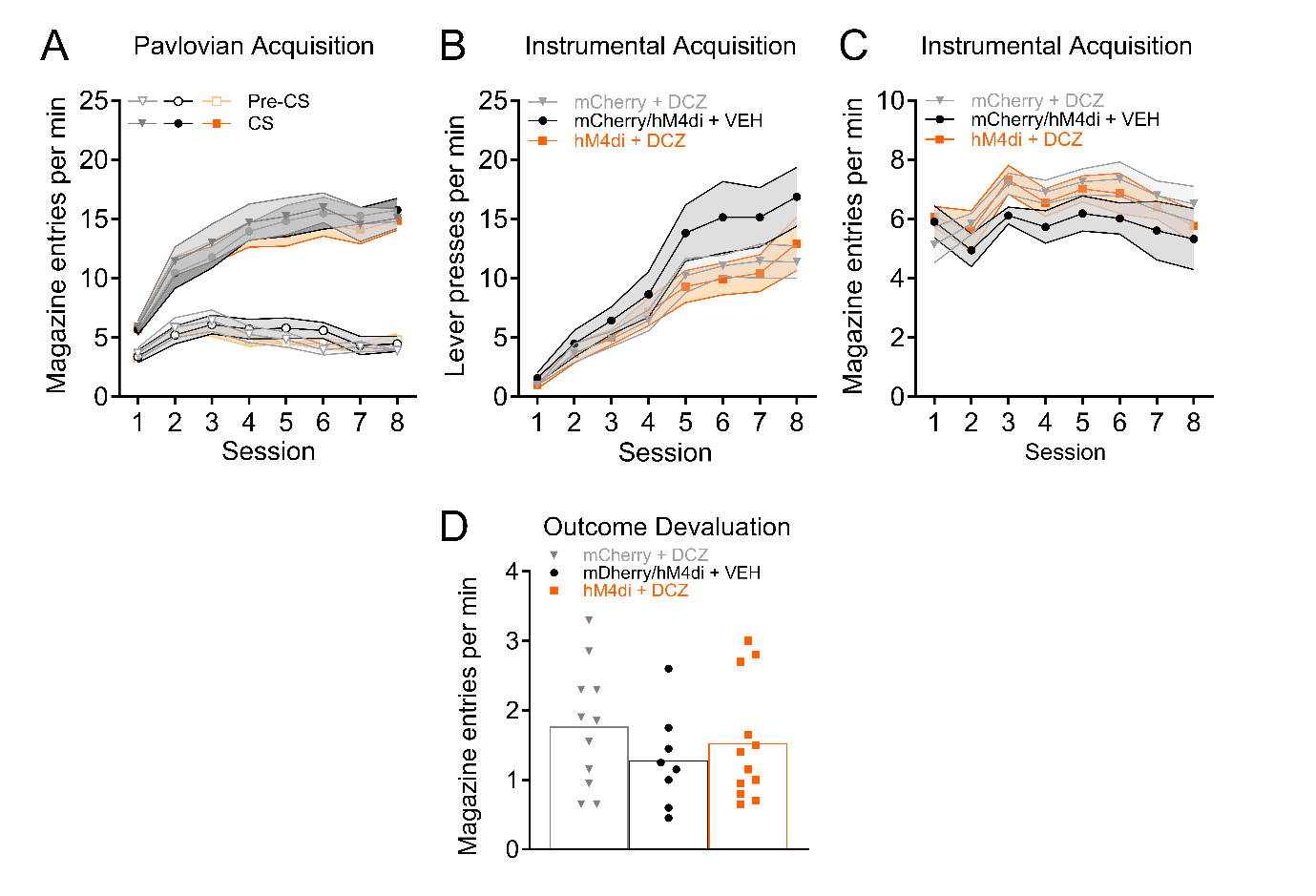
*

**Supplemental Figure 5. Relates to Figure 5. Supplemental behavioural results.** *(A) Magazine entries per min (±SEM) during Pavlovian conditioning, supported by a main effect of CS period (preCS vs CS) F (1,28) = 742.205, p < 0.001, and of Day F (7,196) = 27.685, p < 0.001, and a Day x CS period interaction (preCS vs CS) F (7,196) = 48.789, p < 0.001. No main effect of group or any interactions with group has been detected, Fs < 1, (B) Lever presses per min (±SEM) during instrumental conditioning, supported by a main effect of day F (7, 196) = 67.262, p < 0.001, no main effect of group (F (2, 28) = 1.803, p = 0.183) and no day x group interaction (F (14, 196) = 1.281, p = 0.222), largest F (5.66, 79.3) = 1.281, p = 0.277, for group x session interaction, (C) Magazine entries per min (±SEM) during instrumental conditioning, supported by a main effect of day F (7, 196) = 8.831, p < 0.001, no main effect of group* *(F (2, 28) = 1.827, p = 0.180) and no day x group interaction (F (14, 196) = 0.592, p = 0.870), largest F (10.18, 142.47) = 0.592, p = 0.821, for group x session interaction, (D) Individual data points and mean magazine entries per min during outcome devaluation testing, no group differences in magazine entries on test, F (2, 28) = 0.8223, p = 0.4497.*
